# The landscape of m^6^A regulators in small cell lung cancer: molecular characteristics, immuno-oncology features, and clinical relevance

**DOI:** 10.1186/s12943-021-01408-5

**Published:** 2021-09-27

**Authors:** Chaoqi Zhang, Zhihui Zhang, Zhen Zhang, Yuejun Luo, Peng Wu, Guochao Zhang, Liyan Xue, Qingpeng Zeng, Lide Wang, Zhaoyang Yang, Hua Zeng, Bo Zheng, Fengwei Tan, Qi Xue, Shugeng Gao, Nan Sun, Jie He

**Affiliations:** 1 Department of Thoracic Surgery, National Cancer Center/National Clinical Research Center for Cancer/Cancer Hospital, Chinese Academy of Medical Sciences and Peking Union Medical College, Beijing, 100021 China; 2 Biotherapy Center, The First Affiliated Hospital of Zhengzhou University, Zhengzhou, 450052 Henan China; 3 Department of Pathology, National Cancer Center/ National Clinical Research Center for Cancer/Cancer Hospital, Chinese Academy of Medical Sciences and Peking Union Medical College, Beijing, 100021 China

## Main text

Lung cancer continues to be the world’s most common and deadly cancer [1]. Small cell lung cancer (SCLC) is the most lethal lung cancer and accounts for ~ 15% of total lung cancer cases. SCLC is an aggressive high-grade neuroendocrine tumor and characterized by a short doubling time, rapid growth, and early metastatic spread [2]. Most patients with SCLC rapidly develop drug resistance and their 5-year survival is low (5–6%), even in cases with a good initial response to standard chemotherapy [3]. The addition of immune checkpoint inhibitors to conventional chemotherapy for SCLCs is promising; however, their absolute prolonged benefit is moderate [4]. The complex mechanisms underlying widespread SCLC metastases and recurrence require elucidation to extend the durable benefit of chemotherapy and immunotherapy to more patients.

*N*^6^-methyladenosine (m^6^A), the most abundant and prevalent RNA modification in eukaryotic RNA, is an important component in cancer biology [5]. M^6^A modification—similar to other epigenetic modifications—is a dynamic reversible process regulated by methyltransferases, RNA binding proteins, and demethylases. Accumulating evidence strongly indicates that the m^6^A modification is a novel decisive factor for tumor metastases, recurrence, and therapy resistance, particularly anti-PD-1/PD-L1 monotherapies resistance [6]. Multiple epigenetic abnormalities have been proved tightly associated with cancerous phenotype and aggressiveness in SCLC [3]. However, as the most common RNA epigenetic modification, m^6^A has never been linked to SCLC progression.

This was the first characterization of the landscape of m^6^A regulators—including their molecular characteristics, immuno-oncology features, and clinical relevance. Our data highlight the importance of m^6^A regulators on cancer pathogenesis and shaping of the tumor immune microenvironment (TIME), and lay a rational foundation and buttress for developing therapeutic strategies for targeting the m^6^A modification in patients with SCLC.

## Results and discussion

### The genetic variation, expression patterns, and therapeutic potential of m^6^A regulators in SCLC

**Fig. 1:**
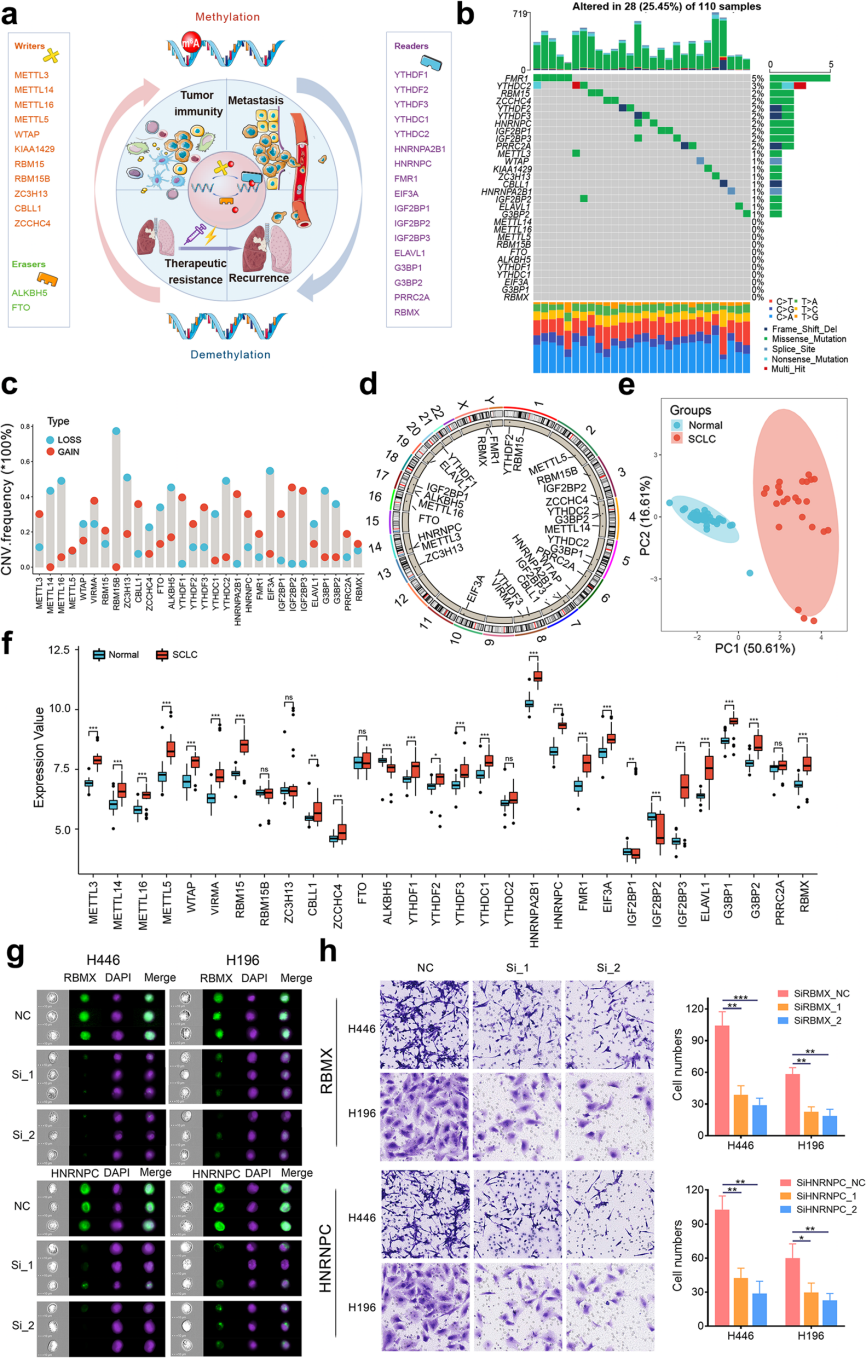
The panorama of genetic variation, expression patterns, and therapeutic potential of m^6^A regulators in small cell lung cancer. **a** Summary of the current knowledge about the dynamic reversible process of m^6^A modification in cancer progression; **b** The mutation frequency of 30 m^6^A regulators in 110 patients with small cell lung cancer from the International Cohort. Each column corresponds to an individual case. The TMB is displayed as the upper bar plot. The right panel shows the mutation frequency and proportion of each variant type for each regulator. The stacked bar plot on the bottom displays the fraction of conversions in each patient. **c** The copy number variation frequency of 30 m^6^A regulators in 53 SCLC cell lines from CCLE. Blue dot, the deletion frequency; Red dot, The amplification frequency. **d** The location of CNV alteration of the m^6^A regulators on 23 chromosomes using data from CCLE. **e** Principal component analysis for the expression profiles of 30 m^6^A regulators to distinguish small cell lung cancer samples from normal lung samples in GSE40275 cohort. There is no intersection between the two subgroups, indicating the small cell lung cancer samples and normal lung samples were well distinguished based on the expression profiles of m^6^A regulators. SCLC samples were marked with red and normal lung samples were marked with blue. **f** The expression detail of 30 m^6^A regulators between normal lung tissues and small cell lung cancer tissues from GSE40275 cohort. Bule box, normal lung samples; red box, small cell lung cancer samples. **g** Image flow cytometry shows the expression of HNRNPC and RBMX in control and knockdown cells (NCIH446 and NCIH196). **h** Transwell migration assays of the migration ability of small cell lung cancer cells (NCIH446 and NCIH196) in the control or knockdown groups. *, **, and *** represent *P* < 0.05, *P* < 0.01, and *P* < 0.001, respectively

We curated a catalog of 30 m^6^A regulators, including 11 writers, two erasers, and 17 readers (Additional file 1: Table S1). The multiple functions of m^6^A modification in cancers are summarized in Fig. 1a. Somatic mutations of 30 m^6^A regulators were explored in 110 SCLC samples. Twenty-eight samples exhibited mutations of m^6^A regulators, with a mutation frequency of 25.5% (Fig. 1b). Of all the regulators, readers showed relatively high mutation frequencies and FMR1 showed the highest mutation frequency. In contrast, no erasers exhibited mutations. We also noted co-occurrent mutations between METTL3 and YTHDC2 and between IGF2BP2 and YTHDC2 (Additional file 2: Figure S1). Then, we investigated the copy number variations (CNVs) of these regulators within 53 SCLC cell lines from the Cancer Cell Line Encyclopedia (CCLE). As shown in Fig. 1c, CNV alterations of m^6^A regulators in SCLC were universal. Most readers (11/17) showed widespread CNV amplification, while all erasers had more loss frequency. The chromosomal CNV alteration mutations are displayed in Fig. 1d. As the genetic alterations of m^6^A regulators are common in SCLC, we should determine if these changes affect expression patterns. The panorama of m^6^A regulator expression in normal lung and SCLC samples were evaluated through principal component analysis, which revealed a dramatically different distribution pattern (Fig. 1e). The expression details of the regulators between normal and SCLC samples are displayed in Fig. 1f. Nearly all the writers and readers were significantly upregulated in SCLCs; nevertheless, eraser expression tended to decline, indicating that SCLC is associated with abundant m^6^A modification. IGF2BP3 has been found at significantly higher levels in multiple solid tumors [7], this research is the first to extend this phenomenon to SCLC. Combining the CNV results with the expression pattern of m^6^A regulators, we infer that CNV alterations may contribute to perturbations in m^6^A regulator expression.

To further investigate the relationship between m^6^A expression profile and SCLC metastasis, we compared the distributions of 30 regulators in 50 SCLC cell lines from CCLE with different pathological origins. Most of the writers and readers showed an upward distribution tendency in metastasis-derived cell lines compared to primary-derived cell lines (Additional file 2: Figure S2), indicating that m^6^A modification may help promote SCLC metastasis. Of note, four regulators—METTL3, HNRNPA2B1, HNRNPC, and RBMX—were significantly upregulated in metastasis-derived cell lines. Could these molecules be therapy targets for metastatic SCLC? We first sought to determine which of the four regulators acted as pan-cancer fitness genes (Additional file 2: Figure S3) [8]. We found that HNRNPC and RBMX were essential genes almost in all different cancer types. Moreover, DepMap data also reminded that HNRNPC and RBMX were necessary for pan-cancer cells (Additional file 2: Figure S4). Therefore, we assumed that HNRNPC and RBMX may also affect the malignant biological behavior of SCLC. We carried out several in vitro and in vivo experiments using two metastasis-derived cell lines and found that HNRNPC and RBMX knockdown drastically inhibited SCLC proliferation and migration and promoted apoptosis (Fig. 1g, h, Additional file 1: Table S2, Additional file 2: Figures S5–S7). We also found the protein expressions of HNRNPC and RBMX were related to SCLC staging in clinical samples (Additional file 2: Figure S8).

### Relationship between m^6^A regulators and cancer pathways and immune characteristics in SCLC

**Fig. 2:**
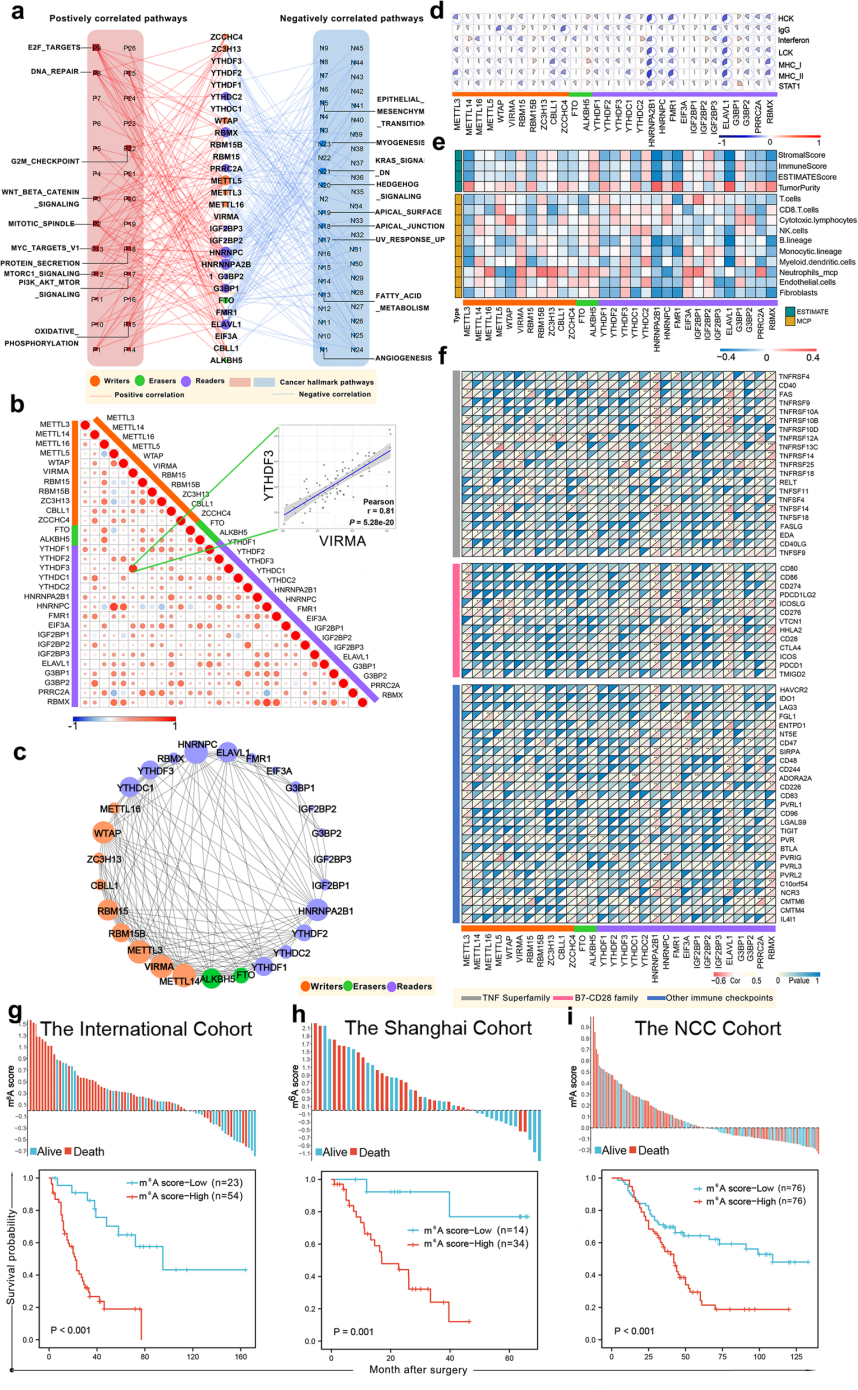
The immuno-oncological features and clinical significance of m^6^A regulators in small cell lung cancer. **a** Network diagram displays the relationship between the selected m^6^A regulators and cancer hallmark-related pathways in small cell lung cancer from the International Cohort. The red and blue lines represent positive and negative correlations, respectively. **b** Correlations between the expression of each m^6^A regulator in small cell lung cancer from the International Cohort. The scatter plot shows the highest correlation coefficient group (VIRMA and YTHDF3, Pearson *R* = 0.81). **c** Protein–protein interactions among the m^6^A regulators. **d** The relationship between m^6^A regulators and inflammatory activities in small cell lung cancer from the International Cohort. **e** Correlation of m^6^A regulators and tumor purity, the fraction of stromal cells, and immune cell populations in small cell lung cancer from the International Cohort. **f** Association between the m^6^A regulators and immune checkpoint profile in small cell lung cancer from the International Cohort. **g** Effects of the 11-regulator based m^6^A score on overall survival of 77 patients from training cohort with RNA-seq data (International Cohort). **h** Validation of m^6^A score in 48 patients from the Shanghai Cohort with RNA-seq data. **i** Validation of the m^6^A score in 152 formalin-fixed paraffin-embedded samples with qPCR data from the NCC Cohort. *, **, and *** represent *P* < 0.05, *P* < 0.01, and *P* < 0.001, respectively

To determine how m^6^A regulators participated in SCLC, we explored correlations between m^6^A regulators and 50 cancer hallmark-related pathways. (Additional file 3: methods). Regulator expression was closely related to different activation statuses of multiple oncogenic pathways (Fig. 2a). With the highest number of connections with activated pathways—including G2M checkpoint, DNA repair, PI3K/AKT/mTOR pathway, et cetera—HNRNPC and METTL5 showed the closest relationship with cancer promotion in SCLC (Fig. 2a, Additional file 2: Figure S9). In contrast, HNRNPA2B1 was correlated with the most inhibited pathways, suggesting that HNRNPA2B1 may be protective for SCLC progression (Additional file 2: Figure S9). In vitro experiments affirmed that METTL5 was a pro-tumoral regulator, and HNRNPA2B1 had no significant effect on SCLC cells (Additional file 2: Figure S10). Of note, m^6^A regulators are well known for their collaboration in cancer progression. We therefore tested the correlation between these 30 regulators in SCLC (Fig. 2b). Several highly correlated relationships were observed between writers and readers, or readers and readers (Additional file 2: Figure S11, *R* > 0.5). These relationships indicated that the cooperation between the writers and readers may promote SCLC progression. Among these relationships, VIRMA and YTHDF3 showed the highest correlation coefficient (Fig. 2b, *R* = 0.81). Additionally, protein–protein interaction networks revealed that these regulators interacted with every single other frequently (Fig. 2c). Notably, writers formed more-frequent connections, which is in line with that the writers are working together as protein complex. Therefore, m^6^A regulator cross-talk and cooperation may participate in SCLC progression.

We also investigated the relationships between regulators and the immune phenotype. Results from seven clusters of metagenes indicated that most regulators were negatively associated with multiple inflammatory actives (Fig. 2d). For instance, ELAVL1 and HNRNPA2B1 showed consistent negative associations with HCK, LCK, MHC-I, STAT1, MHC_ II, Interferon, and Ig G. We also used the ESTIMATE and MCP-counter methods to explore the distributions of stromal and immune cells with different regulators expression patterns (Fig. 2e). Frequent negative correlations were found between the writers and readers with immune-stromal cells, indicating that high m^6^A modification is closely related to immune-desert phenotype in SCLC. Meanwhile, we analyzed the immune checkpoint profiles and found many negative correlations, particularly in writers and readers [especially METTL3, HNRNPA2B1, and ELAVL1 (Fig. 2f)]. These results suggest that m^6^A regulators may be closely related to the heterogeneity and complexity of TIME in SCLCs.

### Clinical significance of the m^6^A regulators in SCLC

Observed connections between m^6^A regulators and immuno-oncological features prompted us to explore the clinical significance of these molecules in SCLC. Most regulators (21/30) exhibited clinical relevance (Additional file 2: Figures S12 and S13a, Additional file 1: Table S3), inspiring us to building a m^6^A regulator-based signature (m^6^A score) for prediction. We assembled three independent cohorts to construct the m^6^A score, including the International Cohort (*n* = 77), the Shanghai Cohort (*n* = 48), and the National Cancer Center (NCC) Cohort (*n* = 152) (Additional file 1: Table S4). A least absolute shrinkage and selection operator model was introduced and a 11-regulator based m^6^A score was established (Additional file 2: Figure S13b, c). The m^6^A score-high patients had significantly worse overall survival (OS) in the International Cohort (Fig. 2g, *P* < 0.001). We confirmed the repeatability and robustness of m^6^A score with the Shanghai cohort (Fig. 2h, *P* = 0.001). To determine if the m^6^A score could be used in clinical practice, we collected 152 SCLC samples with qPCR data from our institute (NCC Cohort) (Additional file 1: Table S5). As expected, the classifier significantly stratified patients into high- vs low-m^6^A score groups for OS (Fig. 2i, *P* < 0.001) and progression-free survival (PFS) (Additional file 2: Figure S14a, *P* = 0.001). The m^6^A score was well verified in different clinical subgroups (Additional file 2: Figure S15) and performed well in patients who received chemotherapy, regardless of cohort (Additional file 2: Figure S14b, c & d, *P* < 0.001). Importantly, after adjusting for clinical and pathologic characteristics, the m^6^A score remained an independent prognostic factor (Additional file 2: Figure S14e). To our best knowledge, the m^6^A score is the first multicenter molecular model for SCLC with a large total sample size. Large-scale investigations are rare because of challenges related to obtaining tumor specimens during standard-of-care treatment regimens. In summary, m^6^A regulators are promising prognostic factors and their future application may help determine the optimal treatment strategy for SCLC.

## Conclusions

This study describes, for the first time, the abnormal expression patterns, specific immuno-oncology features, and clinical relevance of m^6^A regulators in SCLCs. Comprehensive evaluation of m^6^A regulators in SCLCs will help enhance our understanding of tumorigenesis and the remodeled TIME and allow for more effective chemo- and immune-therapeutic options.

## Supplementary Information

**Additional file 1: Supplementary Table S1.** The descriptions of 30 m^6^A regulators enrolled in this study. **Supplementary Table S2.** Primer sequences of cell line samples for qPCR. **Supplementary Table S3.** The optimum cutoff survival analysis of 30 m^6^A regulators in the International Cohort. **Supplementary Table S4.** Clinical characteristics of the patients from multiple institutions. **Supplementary Table S5.** Primer sequences of FFPE samples for qPCR.

**Additional file 2: Supplementary Figure S1.** Co-occurrence of genetic alterations of the m^6^A regulators in small cell lung cancer. **Supplementary Figure S2.** The expression pattern of 30 m^6^A regulators between 18 primary- and 32 metastasis-derived small cell lung cancer cell lines from CCLE. **Supplementary Figure S3.** The essential gene proportion of METTL3 (a), HNRNPA2B1 (b), HNRNPC (c), and RBMX (d) in pan-cancer cell lines. **Supplementary Figure S4.** The landscape of therapeutic potential of HNRNPC and RBMX in pan-cancer cell lines from the DepMap Portal. **Supplementary Figure S5.** HNRNPC is upregulated in small cell lung cancer and promotes cell proliferation and inhibits cell apoptosis. **Supplementary Figure S6.** RBMX is upregulated in small cell lung cancer and promotes cell proliferation and inhibits cell apoptosis. **Supplementary Figure S7.** HNRNPC and RBMX promote SCLC cells metastasis in vivo. **Supplementary Figure S8.** The protein level of HNRNPC and RBMX are positively related to SCLC staging. **Supplementary Figure S9.** The number of m^6^A regulator-related pathways. **Supplementary Figure S10.** Experimental exploration of HNRNPA2B1 and METTL5 in SCLC. **Supplementary Figure S11.** Positive correlations between them^6^A regulators in small cell lung cancer. **Supplementary Figure S12.** Kaplan–Meier survival analysis of SCLCs grouped by the expression of m^6^A regulators in the International Cohort. **Supplementary Figure S13.** The clinical analyze of m^6^A regulators in small cell lung cancer. **Supplementary Figure S14.** The clinical significance of m^6^A regulators in small cell lung cancer. **Supplementary Figure S15.** The performance of the m^6^A score in different clinical subgroups from different cohorts.

**Additional file 3.** Supplementary materials and methods.

## Data Availability

The genetic and expression data of m^6^A regulators in the International Cohort were retrieved from cBioPortal (https://www.cbioportal.org). The expression details of m^6^A regulators in small cell lung cancer cell lines were downloaded from CCLE (https://portals.broadinstitute.org/ccle). The data extracted from GSE40275 and GSE4824 can be downloaded from GEO dataset (https://www.ncbi.nlm.nih.gov/geo/). The other data used and analyzed during this study are available from the corresponding author on reasonable request.

## Abbreviations

SCLC: Small cell lung cancer
m^6^A: *N*^6^-Methyladenosine
TIME: Tumor immune microenvironment
CNV: Copy number variation
CCLE: Cancer Cell Line Encyclopedia
NCC: National Cancer Center
