# Supplementary material for: The landscape of m6A regulators in small cell lung cancer: molecular characteristics, immuno-oncology features, and clinical relevance
Source: Mol Cancer. 2021 Sep 27;20:122. doi: 10.1186/s12943-021-01408-5 (PMC8474928; doi:10.1186/s12943-021-01408-5)
Supplement: Supplementary file 1 — Additional file 1: Supplementary Table S1. The descriptions of 30 m6A regulators enrolled in this study. Supplementary Table S2. Primer sequences of cell line samples for qPCR. Supplementary Table S3. The optimum cutoff survival analysis of 30 m6A regulators in the International Cohort. Supplementary Table S4. Clinical characteristics of the patients from multiple institutions. Supplementary Table S5. Primer sequences of FFPE samples for qPCR. [file 12943_2021_1408_MOESM1_ESM.docx]

**Supplementary Table S1.** The descriptions of 30 m^6^A regulators enrolled in this study.

| Official symbol | Gene ID | Description | Category |
| --- | --- | --- | --- |
| METTL3 | 56339 | Methyltransferase like 3 | Writer |
| METTL14 | 57721 | Methyltransferase like 14 | Writer |
| METTL16 | 79066 | Methyltransferase like 16 | Writer |
| METTL5 | 29081 | Methyltransferase like 5 | Writer |
| WTAP | 9589 | WT1 associated protein | Writer |
| VIRMA | 25962 | Vir like m6A methyltransferase associated | Writer |
| RBM15 | 64783 | RNA binding motif protein 15 | Writer |
| RBM15B | 29890 | RNA binding motif protein 15B | Writer |
| ZC3H13 | 23091 | Zinc finger CCCH-type containing 13 | Writer |
| CBLL1 | 79872 | Cbl proto-oncogene like 1 | Writer |
| ZCCHC4 | 29063 | Zinc finger CCHC-type containing 4 | Writer |
| FTO | 79068 | FTO alpha-ketoglutarate dependent dioxygenase | Eraser |
| ALKBH5 | 54890 | AlkB homolog 5, RNA demethylase | Eraser |
| YTHDF1 | 54915 | YTH N6-methyladenosine RNA binding protein 1 | Reader |
| YTHDF2 | 51441 | YTH N6-methyladenosine RNA binding protein 2 | Reader |
| YTHDF3 | 253943 | YTH N6-methyladenosine RNA binding protein 3 | Reader |
| YTHDC1 | 91746 | YTH domain containing 1 | Reader |
| YTHDC2 | 64848 | YTH domain containing 2 | Reader |
| HNRNPA2B1 | 3181 | Heterogeneous nuclear ribonucleoprotein A2/B1 | Reader |
| HNRNPC | 3183 | Heterogeneous nuclear ribonucleoprotein C | Reader |
| FMR1 | 2332 | FMRP translational regulator 1 | Reader |
| EIF3A | 8661 | Eukaryotic translation initiation factor 3 subunit A | Reader |
| IGF2BP1 | 10642 | Insulin like growth factor 2 mRNA binding protein 1 | Reader |
| IGF2BP2 | 10644 | Insulin like growth factor 2 mRNA binding protein 2 | Reader |
| IGF2BP3 | 10643 | Insulin like growth factor 2 mRNA binding protein 3 | Reader |
| ELAVL1 | 1994 | ELAV like RNA binding protein 1 | Reader |
| G3BP1 | 10146 | G3BP stress granule assembly factor 1 | Reader |
| G3BP2 | 9908 | G3BP stress granule assembly factor 2 | Reader |
| PRRC2A | 7916 | Proline rich coiled-coil 2A | Reader |
| RBMX | 27316 | RNA binding motif protein X-linked | Reader |

**Supplementary Table S2**. Primer sequences of cell line samples for qPCR.

| Gene Name | Forward Primer | Reverse Primer |
| --- | --- | --- |
| HNRNPC | 5'-GTTACCAACAAGACAGATCCTCG-3' | 5'-AGGCAAAGCCCTTATGAACAG-3' |
| RBMX | 5'-TCAAGCAGAGATTACCCAAGTT-3' | 5'-CCACGACCATAATGAGCAAAAT-3' |
| GAPDH | 5'-GGAGCCAAAAGGGTCATCATCTC-3' | 5'-GAGGGGCCATCCACAGTCTTCT-3' |

**Supplementary Table S3**. The optimum cutoff survival analysis of 30 m^6^A regulators in the International Cohort.

| M6A regulators | High expression (*N*) | Low expression (*N*) | *P* value | HR |
| --- | --- | --- | --- | --- |
| METTL3 | 15 | 62 | 0.020 | 0.471 |
| METTL14 | 54 | 23 | 0.004 | 2.364 |
| METTL16 | 36 | 41 | 0.047 | 0.556 |
| METTL5 | 27 | 50 | 0.002 | 2.769 |
| WTAP | 27 | 50 | 0.018 | 2.118 |
| VIRMA | 38 | 39 | 0.286 | 0.735 |
| RBM15 | 64 | 13 | 0.010 | 0.305 |
| RBM15B | 36 | 41 | 0.014 | 0.489 |
| ZC3H13 | 14 | 63 | 0.000 | 0.265 |
| CBLL1 | 28 | 49 | 0.416 | 0.787 |
| ZCCHC4 | 36 | 41 | 0.006 | 2.337 |
| FTO | 69 | 8 | 0.019 | 0.134 |
| ALKBH5 | 57 | 20 | 0.015 | 0.399 |
| YTHDF1 | 49 | 28 | 0.180 | 1.491 |
| YTHDF2 | 64 | 13 | 0.146 | 0.552 |
| YTHDF3 | 45 | 32 | 0.136 | 0.641 |
| YTHDC1 | 62 | 15 | 0.032 | 0.375 |
| YTHDC2 | 37 | 40 | 0.016 | 2.036 |
| HNRNPA2B1 | 38 | 39 | 0.239 | 0.709 |
| HNRNPC | 21 | 56 | 0.008 | 2.669 |
| FMR1 | 11 | 66 | 0.193 | 1.759 |
| EIF3A | 41 | 36 | 0.060 | 0.578 |
| IGF2BP1 | 28 | 49 | 0.001 | 0.400 |
| IGF2BP2 | 38 | 39 | 0.048 | 1.796 |
| IGF2BP3 | 52 | 25 | 0.008 | 0.400 |
| ELAVL1 | 54 | 23 | 0.071 | 0.530 |
| G3BP1 | 21 | 56 | 0.004 | 2.858 |
| G3BP2 | 21 | 56 | 0.002 | 3.328 |
| PRRC2A | 12 | 65 | 0.000 | 0.222 |
| RBMX | 16 | 61 | 0.009 | 2.860 |

**Supplementary Table S4**. Clinical characteristics of the patients from multiple institutions.

| Characteristics | International Cohort (*N*=77) | Shanghai Cohort (*N*=48) | NCC Cohort (*N*=152) |
| --- | --- | --- | --- |
| Age, year |  |  |  |
| <60 | 20 (26.0%) | 27 (56.3%) | 81 (53.3%) |
| ≥60 | 57 (74.0%) | 21 (43.8%) | 71 (46.7%) |
| Sex |  |  |  |
| Male | 54 (70.1%) | 43 (89.6%) | 116 (76.3%) |
| Female | 24 (31.2%) | 5 (10.4%) | 36 (23.7%) |
| Smoking history |  |  |  |
| Yes | 72 (93.5%) | 33 (68.8%) | 93 (61.2%) |
| No | 3 (3.9%) | 15 (31.3%) | 59 (38.8%) |
| NA | 2 (2.6%) | 0 (0.0%) | 0 (0.0%) |
| SCLC staging |  |  |  |
| I | 33 (42.9%) | 8 (16.7%) | 50 (32.9%) |
| II | 14 (18.2%) | 8 (16.7%) | 50 (32.9%) |
| III | 21 (27.3%) | 31 (64.6%) | 52 (34.2%) |
| IV | 9 (11.7%) | 1 (2.1%) | 0 (0.0%) |
| OS state |  |  |  |
| Alive | 29 (37.7%) | 25 (52.1%) | 70 (46.1%) |
| Death | 48 (62.3%) | 23 (47.9%) | 82 (54.0%) |

Data are n (%). NCC, National Cancer Center; NA, not available; SCLC, small cell lung cancer; OS, overall survival.

**Supplementary Table S5**. Primer sequences of FFPE samples for qPCR.

| Gene Name | Forward Primer | Reverse Primer |
| --- | --- | --- |
| PRRC2A | 5'-ACCGATACCCCACTCCTGAT-3' | 5'-CCCACAGGCTCTACTAAGCG-3' |
| IGF2BP1 | 5'-GACCCCTGATGAGAACGACC-3' | 5'-CGGATCTTCCGTTGAGCCAT-3' |
| METTL5 | 5'-GTACGCGGAGTGGCAGAAA-3' | 5'-CAGAGGACGTTGCAGTAGC-3' |
| METTL14 | 5'-AGTTGGGAGCTGAAAGTGCC-3' | 5'-AGCCCTGCAAGTTTCTCTTGT-3' |
| G3BP1 | 5'-CACAAAGACCTCAGCGGGAT-3' | 5'-CTCACGGATTGGTCTGGGTC-3' |
| ZCCHC4 | 5'-CGGAGCTGTCAGTTCTTGGT-3' | 5'-AGCTCATGCAACCTTGGTGT-3' |
| IGF2BP3 | 5'-TGCCACCATTCGGAACATCA-3' | 5'-AATCGACTTCTCAGCAGCCC-3' |
| RBM15B | 5'-GGGAGCATTCGGACCATTGA-3' | 5'-CTCATTTTAGCACAGGCGGC-3' |
| ALKBH5 | 5'-TCAAGCCTATTCGGGTGTCG-3' | 5'-ATCCACTGAGCACAGTCACG-3' |
| YTHDC2 | 5'-CGTGGCTTTGCAAGTCAAGT-3' | 5'-AATCCAATGCCATCCAGCCA-3' |
| IGF2BP2 | 5'-GACAGGTCCTGCTGAAGTCC-3' | 5'-TACCCGAGAGGGTCTCGATG-3' |
| GAPDH | 5'-AAATCAAGTGGGGCGATGCT-3' | 5'-CAAATGAGCCCCAGCCTTCT-3' |
