# Supplementary material for: The landscape of m6A regulators in small cell lung cancer: molecular characteristics, immuno-oncology features, and clinical relevance
Source: Mol Cancer. 2021 Sep 27;20:122. doi: 10.1186/s12943-021-01408-5 (PMC8474928; doi:10.1186/s12943-021-01408-5)
Supplement: Supplementary file 2 — Additional file 2: Supplementary Figure S1. Co-occurrence of genetic alterations of the m6A regulators in small cell lung cancer. Supplementary Figure S2. The expression pattern of 30 m6A regulators between 18 primary- and 32 metastasis-derived small cell lung cancer cell lines from CCLE. Supplementary Figure S3. The essential gene proportion of METTL3 (a), HNRNPA2B1 (b), HNRNPC (c), and RBMX (d) in pan-cancer cell lines. Supplementary Figure S4. The landscape of therapeutic potential of HNRNPC and RBMX in pan-cancer cell lines from the DepMap Portal. Supplementary Figure S5. HNRNPC is upregulated in small cell lung cancer and promotes cell proliferation and inhibits cell apoptosis. Supplementary Figure S6. RBMX is upregulated in small cell lung cancer and promotes cell proliferation and inhibits cell apoptosis. Supplementary Figure S7. HNRNPC and RBMX promote SCLC cells metastasis in vivo. Supplementary Figure S8. The protein level of HNRNPC and RBMX are positively related to SCLC staging. Supplementary Figure S9. The number of m6A regulator-related pathways. Supplementary Figure S10. Experimental exploration of HNRNPA2B1 and METTL5 in SCLC. Supplementary Figure S11. Positive correlations between them6A regulators in small cell lung cancer. Supplementary Figure S12. Kaplan–Meier survival analysis of SCLCs grouped by the expression of m6A regulators in the International Cohort. Supplementary Figure S13. The clinical analyze of m6A regulators in small cell lung cancer. Supplementary Figure S14. The clinical significance of m6A regulators in small cell lung cancer. Supplementary Figure S15. The performance of the m6A score in different clinical subgroups from different cohorts. [file 12943_2021_1408_MOESM2_ESM.docx]

**Supplementary Figures**

**
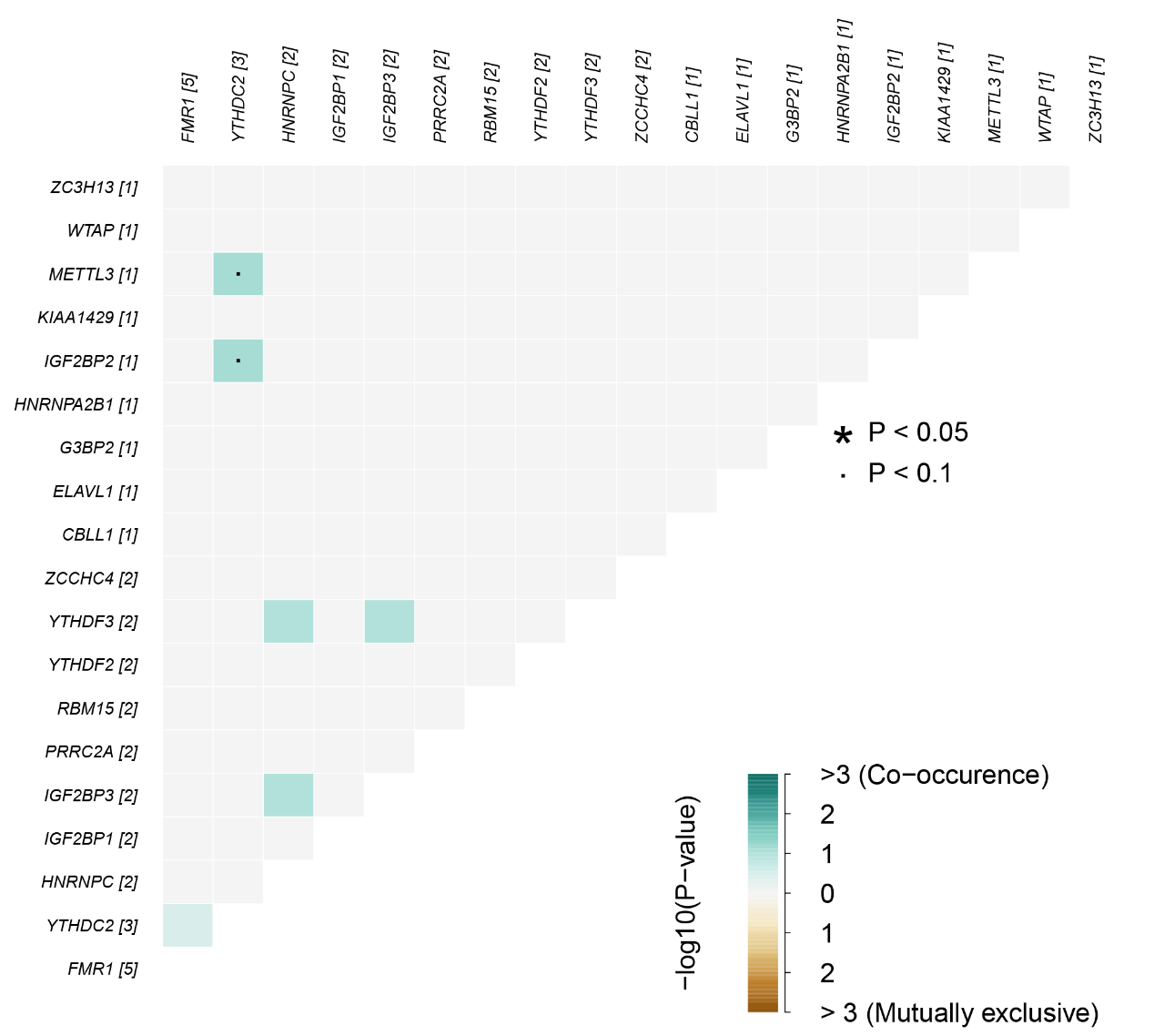
**

**Supplementary Figure S1.** Co-occurrence of genetic alterations of the m^6^A regulators in small cell lung cancer.


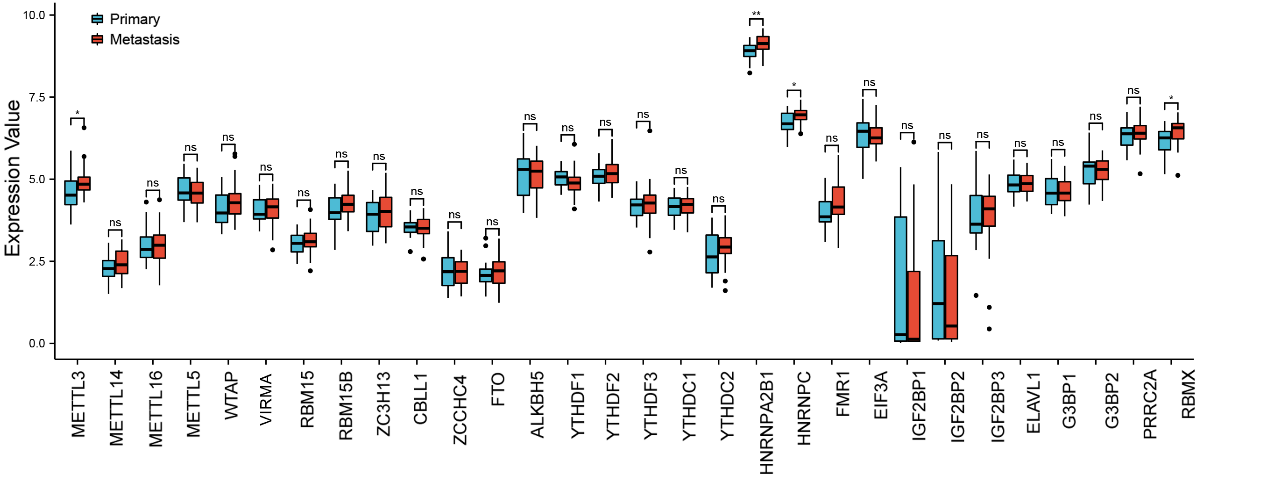


**Supplementary Figure S2.** The expression pattern of 30 m^6^A regulators between 18 primary- and 32 metastasis-derived small cell lung cancer cell lines from CCLE.


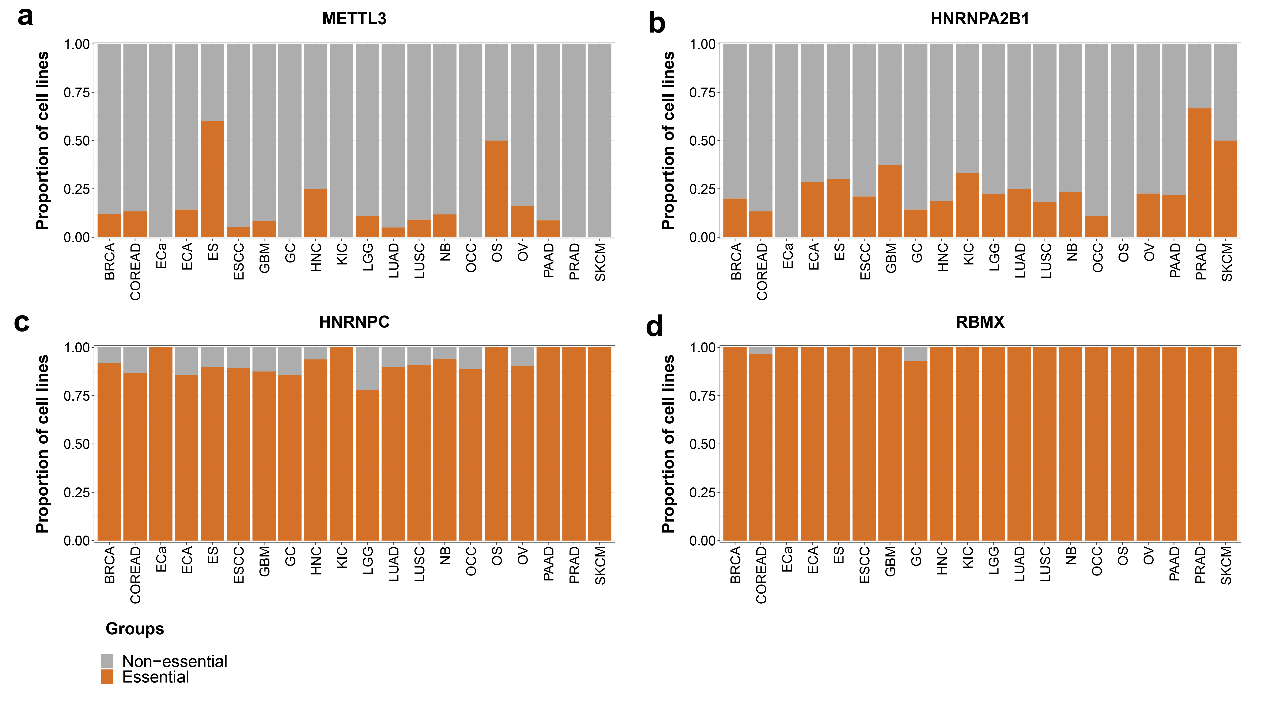


**Supplementary Figure S3.** The essential gene proportion of METTL3 (a), HNRNPA2B1 (b), HNRNPC (c), and RBMX (d) in pan-cancer cell lines. BRCA, Breast Carcinoma; COREAD, Colorectal Carcinoma; ECa, Endometrial Carcinoma; ECA, Esophageal Adenocarcinoma; ES, Ewing`s Sarcoma; ESCC, Esophageal Squamous Cell Carcinoma; GBM, Glioblastoma; GC, Gastric Carcinoma; HNC, Head and Neck Carcinoma; KIC, Kidney Carcinoma; LGG, Low Grade Glioma; LUAD, Lung Adenocarcinoma; LUSC, Squamous Cell Lung Carcinoma; NB, Neuroblastoma; OCC, Oral Cavity Carcinoma; OS; Osteosarcoma; OV, Ovarian Carcinoma; PAAD, Pancreatic Carcinoma; PRAD, Prostate Carcinoma; SKCM, Melanoma.


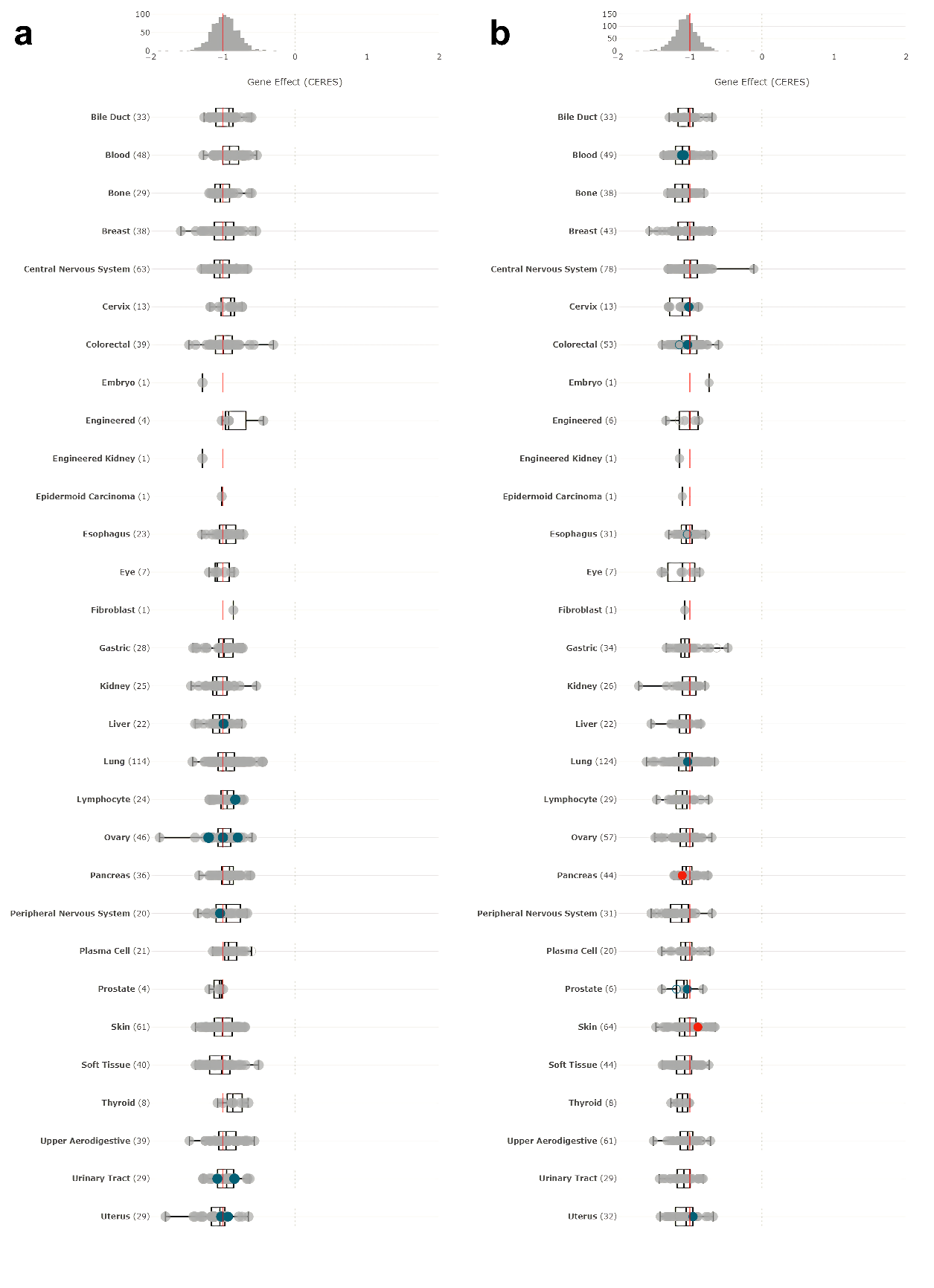


**Supplementary Figure S4.** The landscape of therapeutic potential of HNRNPC and RBMX in pan-cancer cell lines from the DepMap Portal. All the Gene Effect values are less than 0, indicating that HNRNPC and RBMX are pro-tumoral factors in pan-cancer cells.


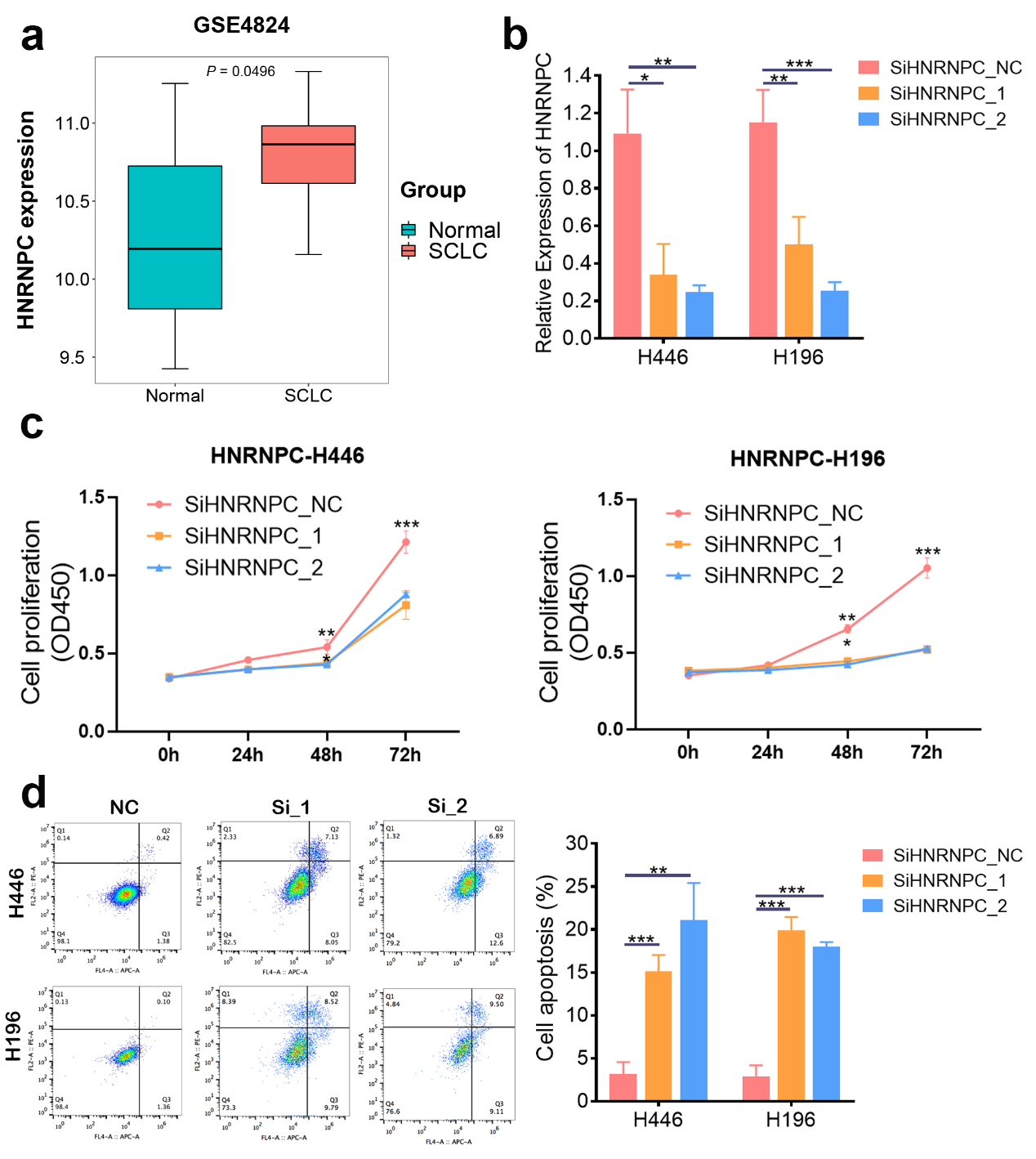


**Supplementary Figure S5.** HNRNPC is upregulated in small cell lung cancer and promotes cell proliferation and inhibits cell apoptosis. a, Compared with normal lung cells, HNRNPC is upregulated in small cell lung cancer cells (cell line data, GSE4824). b, Results of qPCR the knockdown (KD) efficiency of HNRNPC. c, The cell growth rate is evaluated in HNRNPC-KD and control cells. d, Apoptosis is determined in HNRNPC-KD and control cells. *, **, and *** represent *P*<0.05, *P*<0.01, and *P*<0.001, respectively.


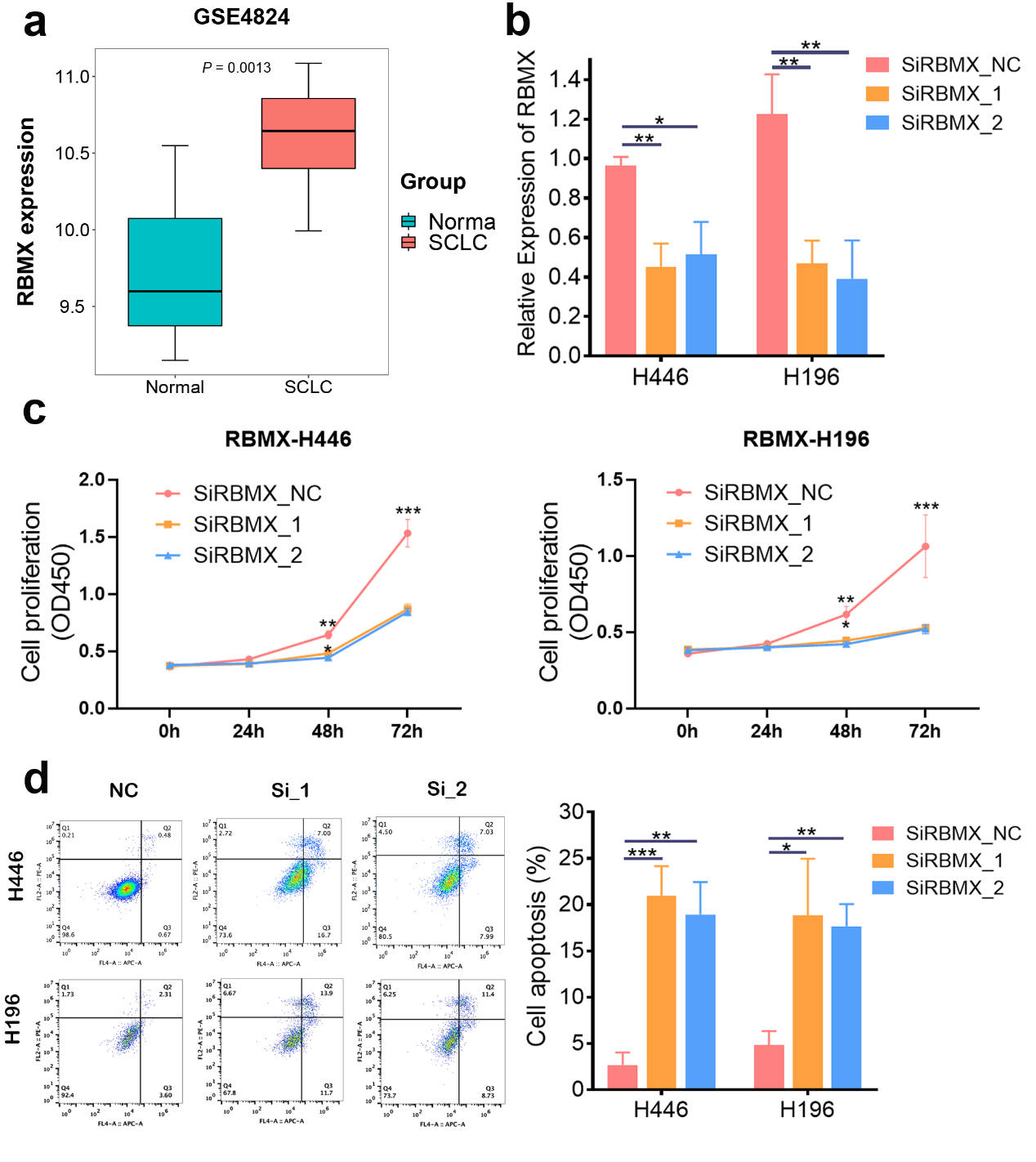


**Supplementary Figure S6.** RBMX is upregulated in small cell lung cancer and promotes cell proliferation and inhibits cell apoptosis. a, Compared with normal lung cells, RBMX is upregulated in small cell lung cancer cells (cell line data, GSE4824). b, Results of qPCR confirm the knockdown (KD) efficiency of RBMX. c, The cell growth rate is evaluated in RBMX-KD and control cells. d, Apoptosis is determined in RBMX-KD and control cells. *, **, and *** represent *P*<0.05, *P*<0.01, and *P*<0.001, respectively.


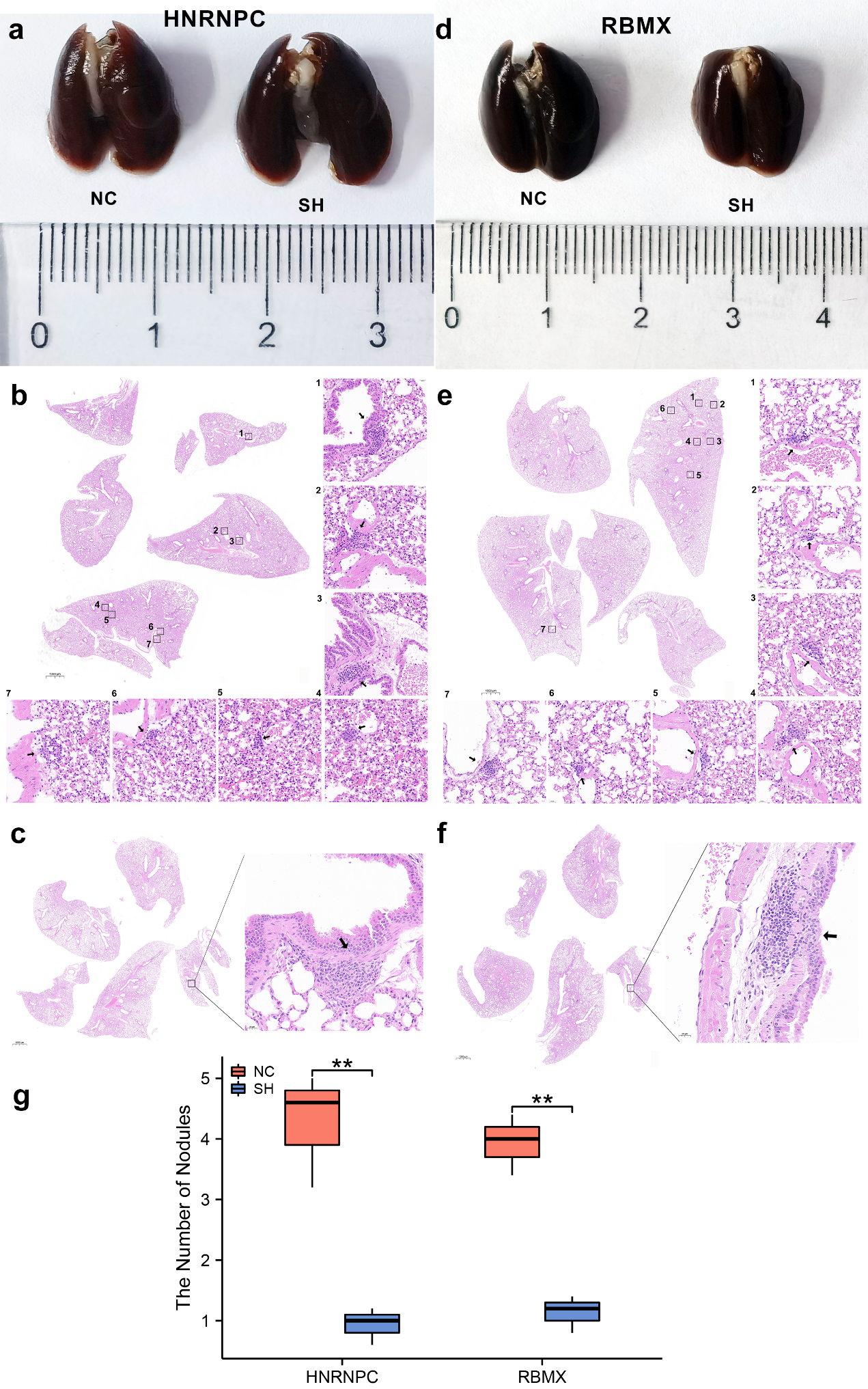


**Supplementary Figure S7.** HNRNPC and RBMX promote SCLC cells metastasis *in vivo*. a, Representative images of isolated lung tissues from the HNRNPC_NC group and the HNRNPC_SH group. b, Representative images of hematoxylin–eosin staining of lung slice from the HNRNPC_NC group. The black boxes indicate the areas of tumor nodules and the black arrows indicate the metastatic cell cluster (upper left panel, ×1; others, ×40). c, Representative images of hematoxylin–eosin staining of lung slice from the HNRNPC_SH group. The black boxes indicate the areas of tumor nodules and the black arrows indicate the metastatic cell cluster (left panel, ×1; right panel, ×40). d, Representative images of isolated lung tissues from the RBMX_NC group and the RBMX_SH group. e, Representative images of hematoxylin–eosin staining of lung slice from the RBMX_NC group. The black boxes indicate the areas of tumor nodules and the black arrows indicate the metastatic cell cluster (upper left panel, ×1; others, ×40). f, Representative images of hematoxylin–eosin staining of lung slice from the RBMX_SH group. The black boxes indicate the areas of tumor nodules and the black arrows indicate the metastatic cell cluster (left panel, ×1; right panel, ×40). g, The number of metastatic nodules in the lungs from different groups. ** represents *P*<0.01.


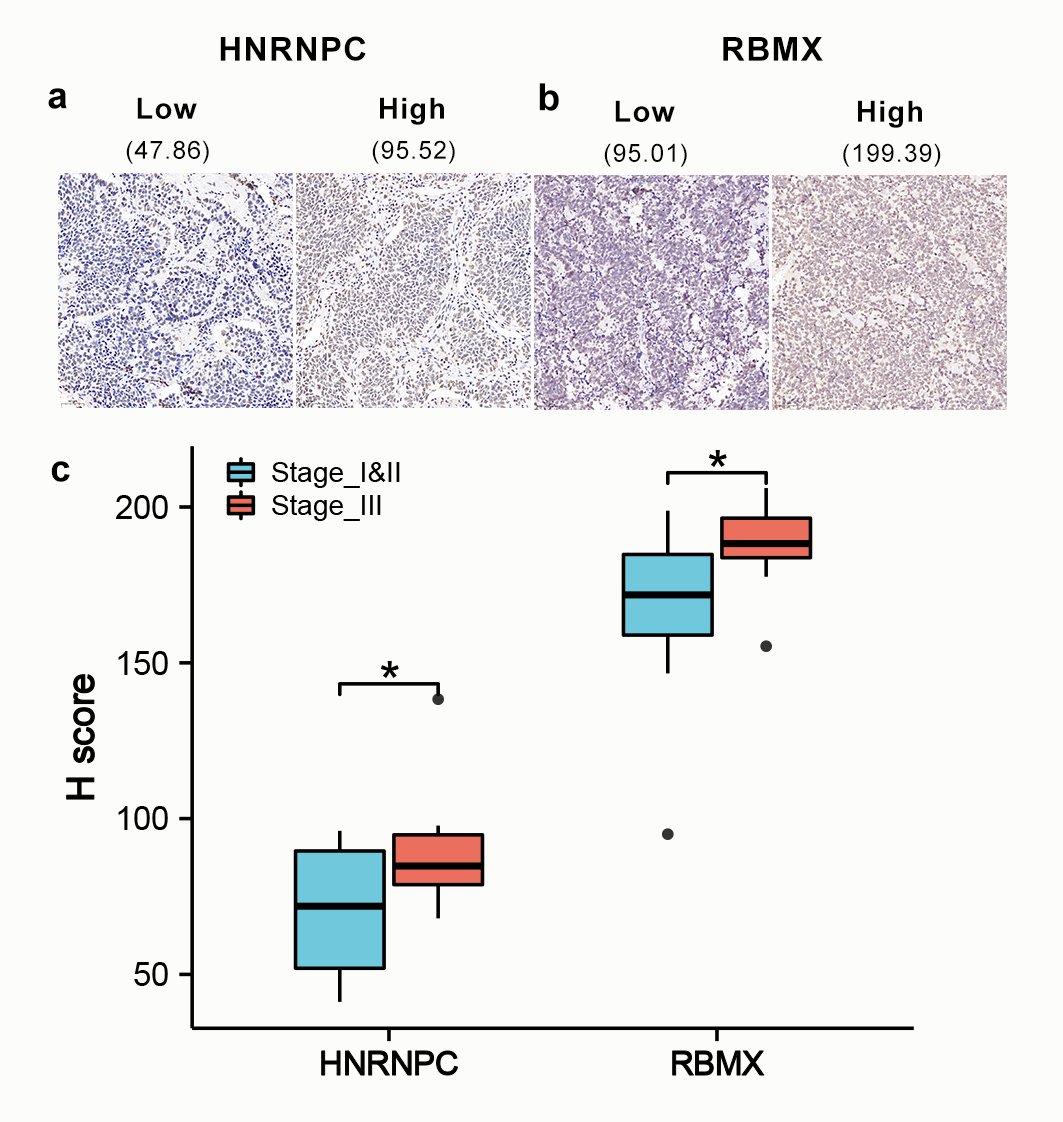


**Supplementary Figure S8.** The protein level of HNRNPC and RBMX are positively related to SCLC staging. a, Representative images of immunohistochemistry results of HNRNPC from SCLC samples (×20, left panel, relatively low expression with H-score=47.86; right panel, relatively high expression with H-score=95.52). b, Representative images of immunohistochemistry results of RBMX from SCLC samples (×20, left panel, relatively low expression with H-score=95.01; right panel, relatively high expression with H-score=199.39). c, The H-scores of HNRNPC and RBMX in SCLC samples (group stage I & II, n=14; group stage III, n=12). * represents *P*<0.05.


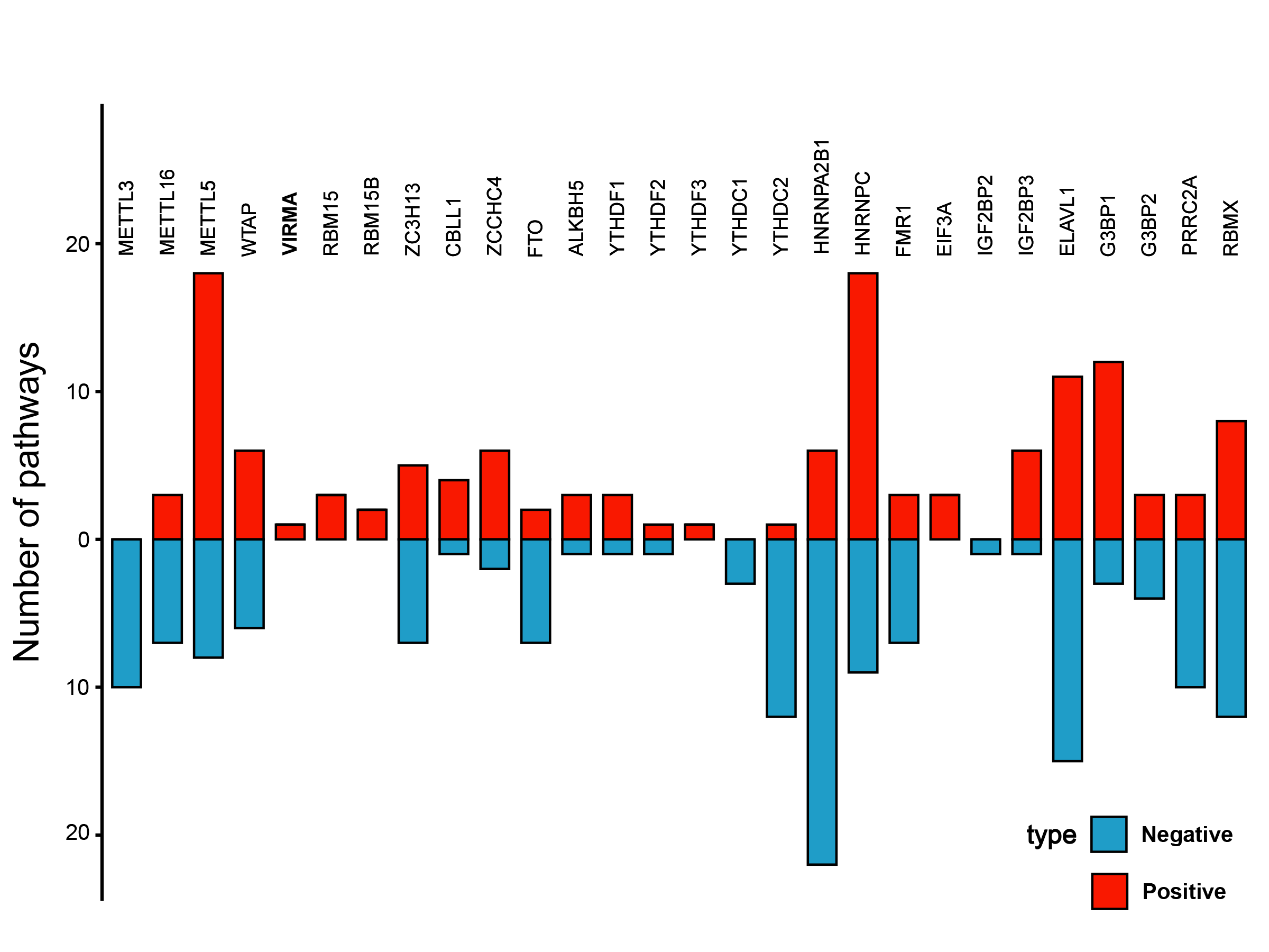


**Supplementary Figure S9.** The number of m^6^A regulator-related pathways. The upper panel (the red bar chart) is for positively related pathways, and the bottom panel (the blue bar chart) is for negatively related pathways.


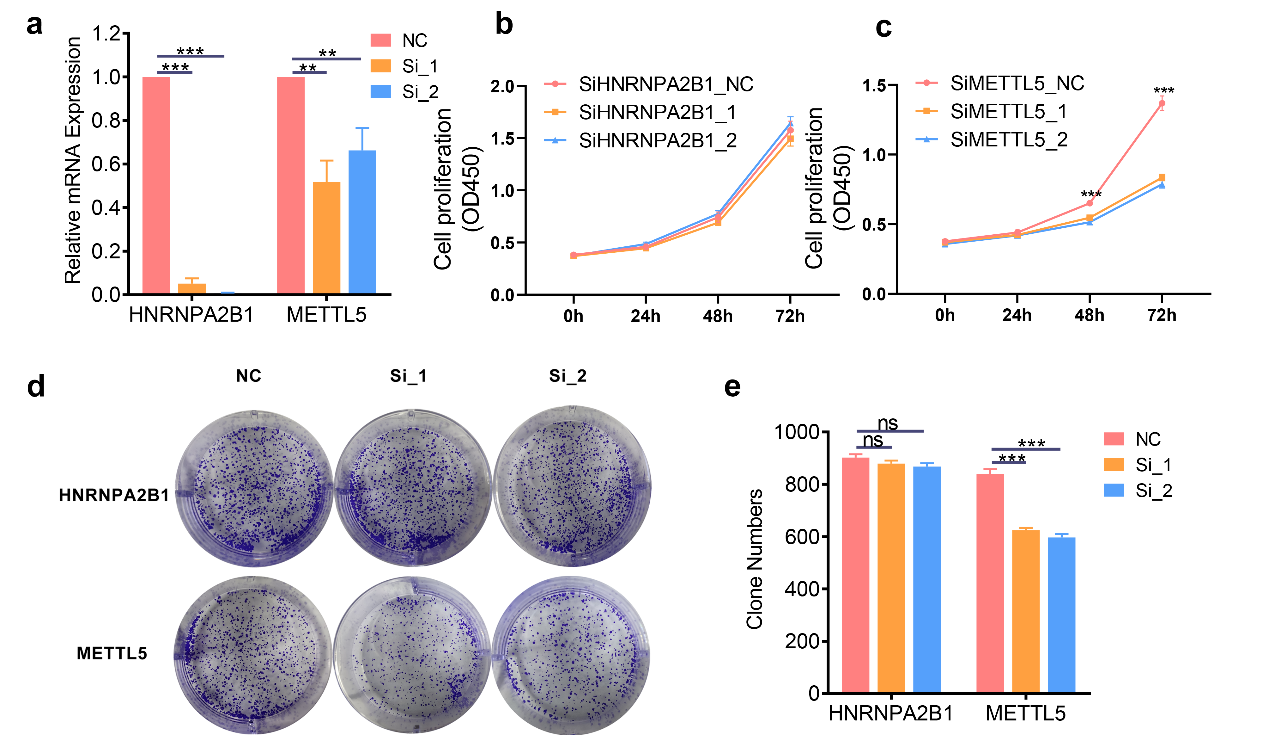


**Supplementary Figure S10.** Experimental exploration of HNRNPA2B1 and METTL5 in SCLC. a, Results of qPCR confirm the knockdown (KD) efficiency of HNRNPA2B1 and METTL5. b, The cell growth rate is evaluated in HNRNPA2B1-KD and control cells. c, The cell growth rate is evaluated in METTL5-KD and control cells. d, Representative images of plate clone formation assay results of KD and control cells. e, The clone numbers in different groups. ** and *** represent *P*<0.01 and *P*<0.001, respectively.


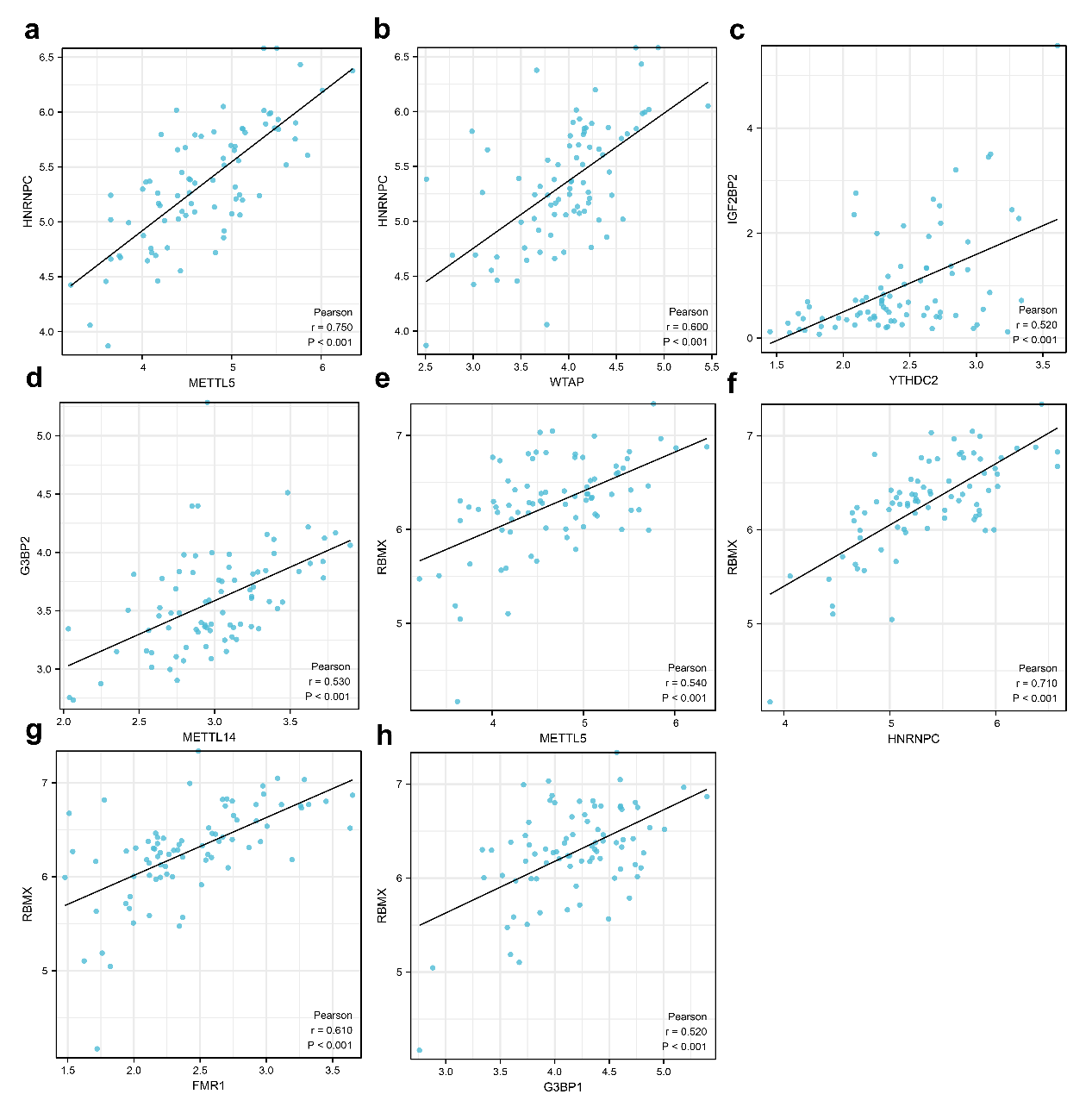


**Supplementary Figure S11.** Positive correlations between the m^6^A regulators in small cell lung cancer. a, METTL5 and HNRNPC. b, WTAP and HNRNPC. c, YTHDC2 and IGF2BP2. d, METTL14 and G3BP2. e, METTL5 and RBMX. f, HNRNPC and RBMX. g, FMR1 and RBMX. h, G3BP1 and RBMX.


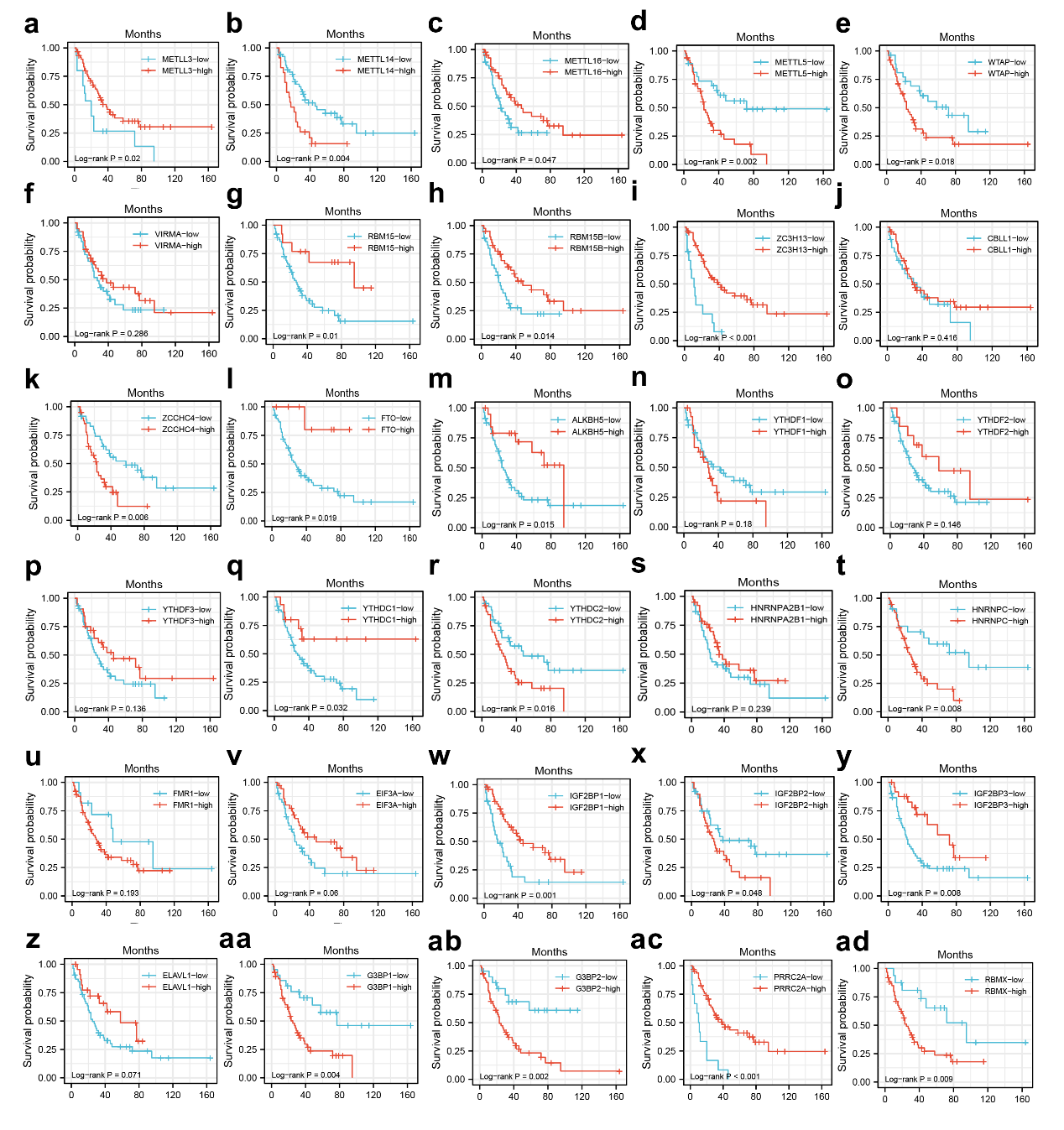
**Supplementary Figure S12.** Kaplan–Meier survival analysis of SCLCs grouped by the expression of m^6^A regulators in the International Cohort, including METTL3 (a) (High expression=62, Low expression=15), METTL14 (b) (High expression=23, Low expression=54), METTL16 (c) (High expression=41, Low expression=36), METTL5 (d) (High expression=50, Low expression=27), WTAP (e) (High expression=50, Low expression=27), VIRMA (f) (High expression=39, Low expression=38), RBM15 (g) (High expression=13, Low expression=64), RBM15B (h) (High expression=41, Low expression=36), ZC3H13 (i) (High expression=63, Low expression=14), CBLL1 (j) (High expression=49, Low expression=28), ZCCHC4 (k) (High expression=41, Low expression=36), FTO (l) (High expression=8, Low expression=69), ALKBH5 (m) (High expression=20, Low expression=57), YTHDF1 (n) (High expression=28, Low expression=49), YTHDF2 (o) (High expression=13, Low expression=64), YTHDF3 (p) (High expression=32, Low expression=45), YTHDC1 (q) (High expression=15, Low expression=62), YTHDC2 (r) (High expression=40, Low expression=37), HNRNPA2B1 (s) (High expression=39, Low expression=38), HNRNPC (t) (High expression=56, Low expression=21), FMR1 (u) (High expression=66, Low expression=11), EIF3A (v) (High expression=36, Low expression=41), IGF2BP1 (w) (High expression=49, Low expression=28), IGF2BP2 (x) (High expression=39, Low expression=38), IGF2BP3 (y) (High expression=25, Low expression=52), ELAVL1 (z) (High expression=23, Low expression=54), G3BP1 (aa) (High expression=56, Low expression=21), G3BP2 (ab) (High expression=56, Low expression=21), PRRC2A (ac) (High expression=65, Low expression=12), and RBMX (ad) (High expression=61, Low expression=16).


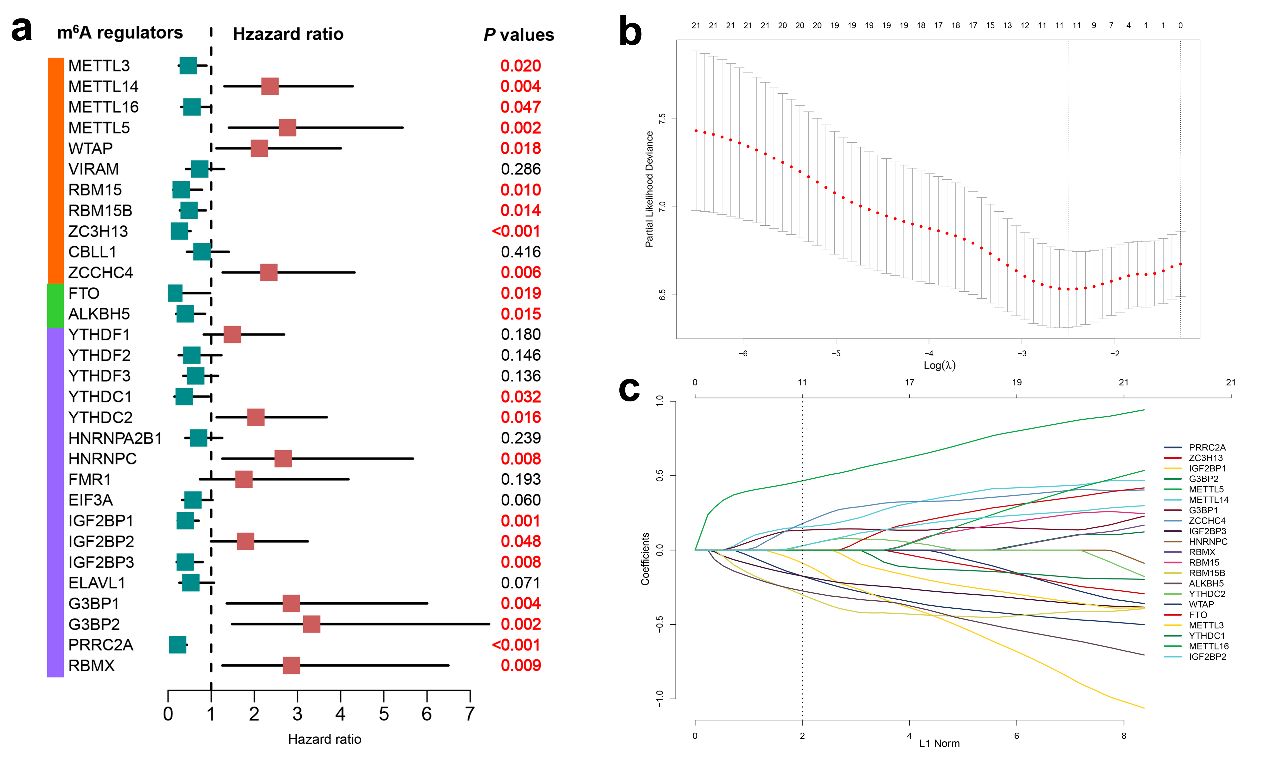


**Supplementary Figure S13.** The clinical analyze of m^6^A regulators in small cell lung cancer. a, The forest plot of the optimum cutoff survival analysis of the m^6^A regulators in small cell lung cancer from the International Cohort. b, The least absolute shrinkage and selection operator is selected to identify the partial likelihood deviance of different numbers of variables and 100-fold cross validation is chosen. The optimal values by the minimum and 1-SE criteria are shown as dotted vertical lines. c, The least absolute shrinkage and selection operator profiles of the significant m^6^A regulators in the International Cohort. Each curve corresponds to a m^6^A regulator.


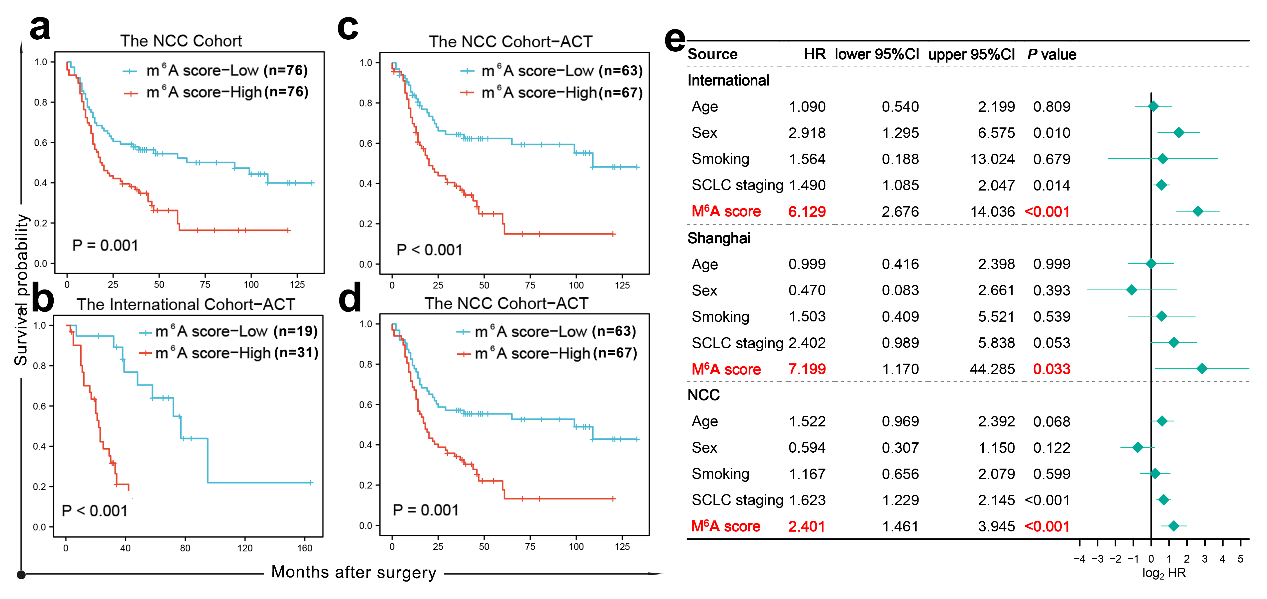


**Supplementary Figure S14.** The clinical significance of m^6^A regulators in small cell lung cancer. a, Survival curve of progression-free survival for patients with high- and low-m^6^A scores from the NCC Cohort. b, Prognostic significance of the m^6^A score in 50 patients with adjuvant chemotherapy from the International Cohort. c, Kaplan–Meier curves of overall survival among 130 patients with adjuvant chemotherapy from the NCC Cohort. d, The m^6^A score predicts the progression-free survival of SCLCs with adjuvant chemotherapy from the NCC Cohort. e, Multivariate Cox regression analysis of clinicopathological factors and m^6^A score for overall survival in patients across multiple centers.


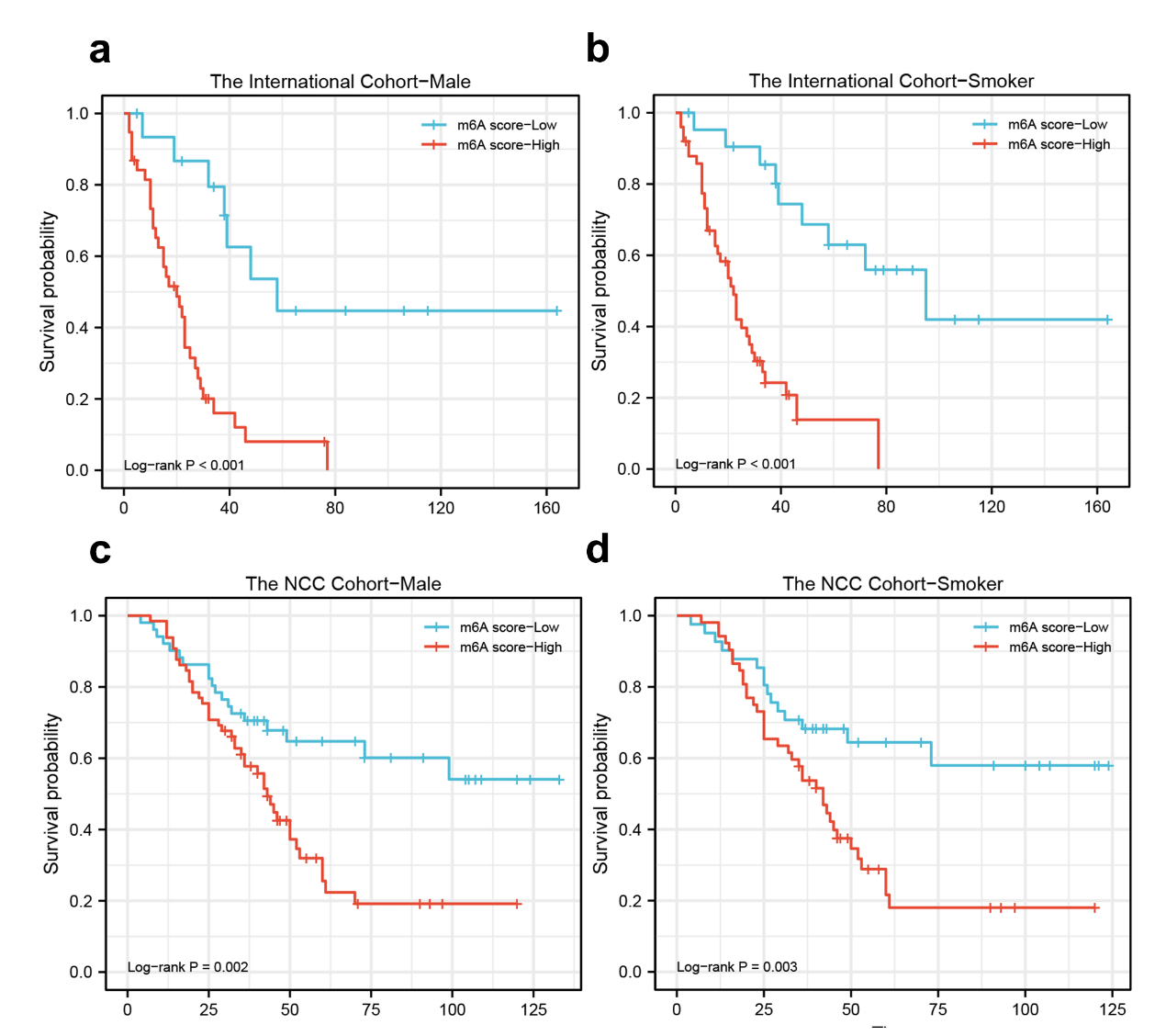


**Supplementary Figure S15.** The performance of the m^6^A score in different clinical subgroups from different cohorts. Kaplan–Meier survival curves of male patients from the International Cohort (a) (m^6^A score high=38, m^6^A score low=16), smokers from the International Cohort (b) (m^6^A score high=50, m^6^A score low=22), male patients from the NCC Cohort (c) (m^6^A score high=65, m^6^A score low=51), and smokers from the NCC Cohort (d) (m^6^A score high=52, m^6^A score low=41) with SCLC classified into high- and low-m^6^A score groups.
