# Supplementary material for: The landscape of m6A regulators in small cell lung cancer: molecular characteristics, immuno-oncology features, and clinical relevance
Source: Mol Cancer. 2021 Sep 27;20:122. doi: 10.1186/s12943-021-01408-5 (PMC8474928; doi:10.1186/s12943-021-01408-5)
Supplement: Supplementary file 3 — Additional file 3. Supplementary materials and methods. [file 12943_2021_1408_MOESM3_ESM.docx]

**Supplementary materials and methods**

**Collection of m^6^A regulators**

We collected 30 m^6^A regulators from recently published literature [1-4], including 11 writers (METTL3, METTL14, METTL16, METTL5, WTAP, VIRMA, RBM15, RBM15B, ZC3H13, CBLL1, and ZCCHC4), two erasers (FTO and ALKBH5), and 17 readers (YTHDF1, YTHDF2, YTHDF3, YTHDC1, YTHDC2, HNRNPA2B1, HNRNPC, FMR1, EIF3A, IGF2BP1, IGF2BP2, IGF2BP3, ELAVL1, G3BP1, G3BP2, PRRC2A, and RBMX).

**Genomic and transcriptomic data** **of m^6^A regulators in clinical samples of small cell lung cancer (SCLC)**

The somatic mutations and expression profile of 30 m^6^A regulators from the International Cohort [5] were downloaded from Cbioportal (<https://www.cbioportal.org/study/summary?id=sclc_ucologne_2015>). The RNA-seq data (Illumina TruSeq) were first log2 transformed and then the mean expression values were recognized as the final expression data when the genes with one more probe were displayed. For analyzing the distributions of regulators between normal and SCLC samples, GSE40275 was selected. The GSE40275 were downloaded from Gene Expression Omnibus (GEO) dataset (<https://www.ncbi.nlm.nih.gov/geo/>) and based on the GPL15974 platform. After acquiring the GPL15974 annotation file (https://www.ncbi.nlm.nih.gov/geo/query/acc.cgi?acc=GPL15974), the probes were mapped according to the annotated detail, and the average RNA expression was chosen for duplicates. The transcriptomic data of significant m^6^A regulators from the Shanghai Cohort were obtained from GSE60052[6]. The normalized log2 transformed data (Illumina HiSeq 2000) were directly downloaded from GEO dataset (<https://www.ncbi.nlm.nih.gov/geo/>).

**Copy number variation and transcriptomic data of m^6^A regulators in cell lines of SCLC**

The copy number variation data across different SCLC lines were gathered from the Broad Institute Cancer Cell Line Encyclopedia (CCLE)[7]. The expression pattern of m^6^A regulators between primary- and metastasis-derived cell lines were collected from CCLE with RNA-seq data (Illumina HiSeq, log2 transformed RPKM). The distributions of HNRNPC and RBMX in normal lung cancer cell lines and SCLC cell lines were extracted from GSE4824 download from GEO dataset (<https://www.ncbi.nlm.nih.gov/geo/>).

**Cancer hallmark-related pathways of SCLC**

To calculate the relevance of m^6^A regulators and the activity of oncogenic pathway activity in SCLC, the well-defined 50 cancer hallmark-related pathways gene sets were collected from Molecular Signature Database of Gene Set Enrichment Analysis (Hallmark gene sets, <http://www.gsea-msigdb.org/gsea/msigdb>). The expression data of these genes were extracted from the International Cohort. Gene Set Variation Analysis (GSVA) was used to calculate the enrichment score of each pathway[8]. Then, a Pearson correlation analysis was conducted to calculate the correlation coefficient between the regulators and pathways. Only the Pearson |R|>0.3 and *P*<0.001 was filtered out and displayed as significant.

**Protein-protein interaction analysis of m^6^A regulators**

The human protein-protein interactions among different m^6^A regulators were derived from the STRING interaction database (<https://string-db.org/>) [9]. Then the visualization of this interaction was realized by Cytoscape[10].

**The immune phenotype of SCLC**

Three aspects were included to explore the relationship between the m^6^A regulators and the immune phenotype. First, 7 clusters of metagenes represent different types of inflammation and immune responses were chosen [11]. Then, GSVA was also used before the correlation analysis. The second aspect, stromal and immune cells were calculated using ESTIMATE (Estimation of Stromal and Immune cells in Malignant Tumor tissues using Expression data)[12] and MCP-counter (Microenvironment Cell Populations-counter) [13] methods. Based on the tissue-derived gene expression data, ESTIMATE was used to predict tumor purity and stromal and immune scores. MCP-counter was used to estimate the abundance of eight tumor-infiltrating leukocytes and two stromal cell populations based on the tissue-derived transcriptomic data. The third aspect was the immune checkpoint profile, including the B7-CD28 family, tumor necrosis factor (TNF) superfamily, and some other immune checkpoints [14-16].

**Patients and tissue specimens**

The protocol of this study conformed to the ethical guidelines of the Declaration of Helsinki and approved by the Ethics Committee of our institute (the National Cancer Center/Cancer Hospital of the Chinese Academy of Medical Sciences). Considering the retrospective nature of our study, the requirement for informed consent was waived by the Ethics Committee. All the patient-derived data were anonymously analyzed.

The 152 SCLC samples from our institute (the National Cancer Center, NCC) were formalin-fixed paraffin-embedded (FFPE) archived samples collected from surgery. All these patients were initially diagnosed as primary SCLC, with no evidence of other tumors, at the NCC from January 2009 to November 2017. The diagnosis of SCLC was reviewed by two pathologists (Liyan Xue and Zhaoyang Yang) by pathological histology diagnosis through hematoxylin and eosin (H&E) staining, and neuroendocrine markers (chromogranin A and synaptophysin) through immunohistochemistry. No patients received neoadjuvant therapy and 130 patients received adjuvant chemotherapy. The clinical and pathological features of these patients are summarized in Table S4. We defined the day of surgery as the day of recurrence, metastasis, or last follow-up as the RFS and day of surgery to the day of death or last follow as the OS.

**RNA isolation and qPCR**

For the RNA isolation of FFPE samples, only tissue biopsies that were at least 70% tumor cells were selected and ~30 μm sections were cut from the FFPE blocks. The FFPE-derived RNA extraction was realized through using the Ambion RecoverAll Total Nucleic Acid Isolation Kit for FFPE (ThermoFisher, Waltham, MA, USA) according to the manufacturer’s instructions. For RNA isolation of cell lines, total RNA was extracted using TRIzol reagent (Invitrogen, Carlsbad, CA). Then, the quality and quantity of isolated RNA were measured by NanoDrop 2000C spectrophotometer (Thermo Scientific, Waltham, MA, USA). Only RNA with an A260/A280 ratio of ≥1.8 was selected. Next, cDNA was reverse transcribed according to the FastKing RT Kit（With gDNase）(KR116) manufacturer’s instructions (Tiangen, Beijing, China). Finally, qPCR was conducted with SYBR Green (#208054, Qiagen, Dusseldorf, Germany), and the housekeeping gene, GAPDH, was selected as an internal control. The analysis of relative m^6^A regulator gene expression was calculated using the 2^−ΔΔCt^ method. Commercially available mRNA primers for FFPE samples and cell line samples for qPCR were summarized in Table S5 and S2, respectively.

**Immunohistochemistry staining and the H-score system**

We obtained 1.0-mm core biopsies from the most representative tumor area of each sample to build the tissue microarrays (TMAs). The TMA samples were subjected to immunohistochemical analysis using standard protocols. The TMAs were incubated with two primary antibodies—anti-HNRNPC (1:1000 dilution, Abcam, ab10294) and anti-RBMX (1:500 dilution, Abcam, ab190352)—followed by incubation with secondary antibodies. Using a digital pathology image analysis, the regulators’ staining levels were estimated using a histologic score (H-score) system [17]. Each sample’s H-score value ranged from 0 (no staining) to 300 (maximum immunoreactivity). The H-score was calculated according to immunostaining intensity and the corresponding percentage. Specifically speaking, the intensities were assessed as 0–3: 0, negative staining; 1, light staining; 2, moderate staining; 3, strong staining. Then, the H-score was conducted by a formula, H-score= 3 × (% at 3) + 2 × (% at 2) + 1 × (% at 1).

**Fitness genes and DepMap data**

The fitness genes were identified from a previous study [18]. Fitness genes were defined as *genes required for cell growth or viability* in the cancer cell lines. DepMap Portal (<https://depmap.org/portal/>) data was used to further validated the essential roles of HNRNPC and RBMX in cancer cells.

**RNA interference**

For HNRNPC, RBMX, METTL5, and HNRNPA2B1 knockdown, commercially available siRNA designed and purchased from Syngentech Company (Beijing, China) was transfected into NCIH196 and NCIH446 cells using Lipofectamine 3000 (Invitrogen, Carlsbad, CA, USA), with a final concentration of 20 nM, according to the manufacturer’s instructions.

**Cell viability and apoptosis assay**

The cell proliferation rate was conducted using Cell Counting Kit-8 (CCK-8; Dojindo, Japan) based on the manufacturer’s instructions and was detected every 24h by measuring the absorbance at 450 nm with a plate reader (VARIOSKAN LUX, Thermo Scientific, USA). Flow cytometric was used to evaluate apoptosis. After washing two times with ice-cold PBS, the cells were then suspended in the Annexin V-binding buffer with the concentration of 10^6^ cells/ml. After incubating with AlexaFluor 647 Annexin V (Biolegend, USA) for 15 min at 4 °C in the dark, then PI (Sigma, USA) was added. After that, samples were immediately analyzed by flow cytometry (FACSCanto II, BD, USA).

**Imaging Flow Cytometry**

To verify the efficiency of RNA interference, image flow cytometry was used to detected the protein level of HNRNPC and RBMX in SCLC cell lines. Before co-incubated with the primary antibodies—anti-HRNPC (ab10294, Abcam, Cambridge, UK), anti-RBMX (ab190352, Abcam, Cambridge, UK)—the cells were first harvested, fixed with 4% paraformaldehyde for 15 min at room temperature, and permeabilized with 0.1% saponin (Sigma, USA). The secondary fluorochrome-conjugated antibodies were then used (ThermoFisher, A32766 & A32754). Subsequently, the tumor cells were counter-stained with DAPI. Finally, the cells were detected using ImageStream MKII system (Amnis, Germany) [19].

***In vivo* animal experiments**

To explore the therapeutic potential of targeting HNRNPC and RBMX in SCLC, *in vivo* lung colonization assay was conducted. Firstly, stable cell lines (NCIH446) with HNRNPC or RBMX knock-down were constructed. We purchased 12, 6-8 weeks old female NOD/SCID-beige mice from Huafukang Bioscience (Beijing, China). The mice were housed under standard conditions at our institution’s animal research facility. Twelve mice were divided into four groups (HNRNPC-NC, HNRNPC-SH, RBMX-NC, RBMX-SH, n=3/group) and tail-vein injections were administered with 1×10^6^ NCIH446 cells transfected with corresponding plasmids. Approximately 8–9 weeks later, the mice were sacrificed using CO_2_ anesthesia, and their lungs were separated. The lung tissues were soaked with picric acid and embedded in paraffin for H&E staining. Representative nodule images were collected after H&E staining. All procedures were approved by our institution’s Animal Care and Use Committee.

**Cell migration assay**

Cell migration assay was conducted using a Transwell system with a 24-well inserted plate (5.0-μm pore size) following the manufacturer's instructions. The suspension of SCLC cell lines (2 × 10^5^ cells/well) in 200 μL FBS-free RMPI-1640 was added to the upper chamber. Simultaneously, 600 μL RMPI-1640 (20% FBS) was added to the lower chamber. Twenty-four hours later, cells attaching on the lower surface were stained with 0.1% crystal violet for 15 min.

**M^6^A score system construction and statistical analysis**

To filter out the significant m^6^A regulators from SCLC samples, optimum cutoff survival analysis was conducted, which was realized through the “surv_cutpoint” function of the “survminer” R package[14]. This process produced 21 significant regulators, including 10 regulators (METTL14, METTL5, WTAP, ZCCHC4, YTHDC2, HNRNPC, IGF2BP2, G3BP1, G3BP2, and RBMX) as risky factors (HR>1), and 11 regulators (METTL3, METTL16, RBM15, RBM15B, ZC3H13, FTO, ALKBH5, YTHDC1, IGF2BP1, IGF2BP3, and PRRC2A) as protective factors (HR<1). Then, a popular method for variable selection—the least absolute shrinkage and selection operator (LASSO) method for variable selection in a Cox regression model[20]—was used to select the most useful m^6^A regulators with the R package ‘‘glmnet’’. Then, the m6A score system for patients with SCLC was built by considering the regulators’ expression and correlation-estimated Cox regression coefficients: m^6^A score = (PRRC2A × -0.1627) + (IGF2BP1 × -0.0702) + (METTL5 × 0.4571) + (METTL14 × 0.151) + (G3BP1 × 0.1318) + (ZCCHC4 × 0.1609) + (IGF2BP3 × -0.1672) + (RBM15B × -0.2846) + (ALKBH5 × -0.2664) + (YTHDC2 × 0.0212) + (IGF2BP2 × 0.0184). Kaplan–Meier curve analysis with a two-tailed log-rank test was used to evaluate the prognostic significance of the m^6^A score in different cohorts and subgroups. To determine whether the m^6^A score is an independent prognostic factor across multiple centers, the R package “‘survival” was used to conduct the multivariate Cox regression analysis. The Mann–Whitney *U*-test was introduced to calculate the between-group differences for m^6^A regulators. R software (version 3.5.1) (https://www.r-project.org) was used for the data processing and analysis. A significant difference in all statistical methods of this study was considered if the *P* value was less than 0.05.

**References**

1. Li Y, Xiao J, Bai J, Tian Y, Qu Y, Chen X, Wang Q, Li X, Zhang Y, and Xu J, Molecular characterization and clinical relevance of m(6)A regulators across 33 cancer types. Mol Cancer, 2019. 18(1):137.

2. Liu J, Harada BT, and He C, Regulation of Gene Expression by N(6)-methyladenosine in Cancer. Trends Cell Biol, 2019. 29(6):487-499.

3. Huang H, Weng H, and Chen J, m(6)A Modification in Coding and Non-coding RNAs: Roles and Therapeutic Implications in Cancer. Cancer Cell, 2020. 37(3):270-288.

4. Nombela P, Miguel-López B, and Blanco S, The role of m(6)A, m(5)C and Ψ RNA modifications in cancer: Novel therapeutic opportunities. Mol Cancer, 2021. 20(1):18.

5. George J, Lim JS, Jang SJ, Cun Y, Ozretić L, Kong G, Leenders F, Lu X, Fernández-Cuesta L, Bosco G, et al., Comprehensive genomic profiles of small cell lung cancer. Nature, 2015. 524(7563):47-53.

6. Jiang L, Huang J, Higgs BW, Hu Z, Xiao Z, Yao X, Conley S, Zhong H, Liu Z, Brohawn P, et al., Genomic Landscape Survey Identifies SRSF1 as a Key Oncodriver in Small Cell Lung Cancer. PLoS Genet, 2016. 12(4):e1005895.

7. Ghandi M, Huang FW, Jané-Valbuena J, Kryukov GV, Lo CC, McDonald ER, 3rd, Barretina J, Gelfand ET, Bielski CM, Li H, et al., Next-generation characterization of the Cancer Cell Line Encyclopedia. Nature, 2019. 569(7757):503-508.

8. Hänzelmann S, Castelo R, and Guinney J, GSVA: gene set variation analysis for microarray and RNA-seq data. BMC Bioinformatics, 2013. 14:7.

9. Szklarczyk D, Franceschini A, Wyder S, Forslund K, Heller D, Huerta-Cepas J, Simonovic M, Roth A, Santos A, Tsafou KP, et al., STRING v10: protein-protein interaction networks, integrated over the tree of life. Nucleic Acids Res, 2015. 43(Database issue):D447-52.

10. Shannon P, Markiel A, Ozier O, Baliga NS, Wang JT, Ramage D, Amin N, Schwikowski B, and Ideker T, Cytoscape: a software environment for integrated models of biomolecular interaction networks. Genome Res, 2003. 13(11):2498-504.

11. Rody A, Holtrich U, Pusztai L, Liedtke C, Gaetje R, Ruckhaeberle E, Solbach C, Hanker L, Ahr A, Metzler D, et al., T-cell metagene predicts a favorable prognosis in estrogen receptor-negative and HER2-positive breast cancers. Breast Cancer Res, 2009. 11(2):R15.

12. Yoshihara K, Shahmoradgoli M, Martínez E, Vegesna R, Kim H, Torres-Garcia W, Treviño V, Shen H, Laird PW, Levine DA, et al., Inferring tumour purity and stromal and immune cell admixture from expression data. Nat Commun, 2013. 4:2612.

13. Becht E, Giraldo NA, Lacroix L, Buttard B, Elarouci N, Petitprez F, Selves J, Laurent-Puig P, Sautès-Fridman C, Fridman WH, et al., Estimating the population abundance of tissue-infiltrating immune and stromal cell populations using gene expression. Genome Biol, 2016. 17(1):218.

14. Zhang C, Zhang Z, Sun N, Zhang Z, Zhang G, Wang F, Luo Y, Che Y, and He J, Identification of a costimulatory molecule-based signature for predicting prognosis risk and immunotherapy response in patients with lung adenocarcinoma. Oncoimmunology, 2020. 9(1):1824641.

15. Mezzadra R, Sun C, Jae LT, Gomez-Eerland R, de Vries E, Wu W, Logtenberg MEW, Slagter M, Rozeman EA, Hofland I, et al., Identification of CMTM6 and CMTM4 as PD-L1 protein regulators. Nature, 2017. 549(7670):106-110.

16. Sadik A, Somarribas Patterson LF, Öztürk S, Mohapatra SR, Panitz V, Secker PF, Pfänder P, Loth S, Salem H, Prentzell MT, et al., IL4I1 Is a Metabolic Immune Checkpoint that Activates the AHR and Promotes Tumor Progression. Cell, 2020. 182(5):1252-1270.e34.

17. Yao B, Gui T, Zeng X, Deng Y, Wang Z, Wang Y, Yang D, Li Q, Xu P, Hu R, et al. PRMT1-mediated H4R3me2a recruits SMARCA4 to promote colorectal cancer progression by enhancing EGFR signaling. Genome Med. 2021;13(1):58.

18. Behan FM, Iorio F, Picco G, Gonçalves E, Beaver CM, Migliardi G, Santos R, Rao Y, Sassi F, Pinnelli M, et al. Prioritization of cancer therapeutic targets using CRISPR-Cas9 screens. Nature. 568(7753):511-516.

19. Ping Y, Li F, Nan S, Zhang D, Shi X, Shan J, and Zhang Y, Augmenting the Effectiveness of CAR-T Cells by Enhanced Self-Delivery of PD-1-Neutralizing scFv. Front Cell Dev Biol, 2020. 8:803.

20. Tibshirani R, The lasso method for variable selection in the Cox model. Stat Med, 1997. 16(4):385-95.
